# Addressing complex autofluorescence signatures in solid tissue samples to enhance full spectrum flow cytometry of non-immune cells

**DOI:** 10.1016/j.bbrep.2026.102845

**Published:** 2026-10-17

**Authors:** Christina Gkantsinikoudi, Manuela Terranova-Barberio, Neil Dufton

**Affiliations:** a Centre for Microvascular Research, William Harvey Research Institute, Queen Mary University of London, EC1M 6BQ, United Kingdom; b Flow Cytometry Core Facility, Barts Cancer Institute, Queen Mary University of London, London, EC1M 6BQ, United Kingdom

**Keywords:** Full spectrum flow cytometry, High-dimensional single-cell analysis, Endothelial heterogeneity, Endothelial cell phenotyping, Inflammation, Fibrosis, Autofluorescence

## Abstract

FSFC is an emerging technology that can greatly enhance our understanding of the single-cell proteomic landscape. We demonstrate that this application is applicable to cells derived from solid tissues which have traditionally been hampered by their complex autofluorescence signatures and lack of optimized tools for non-immune cells. We present a protocol and discuss key controls that minimize the impact of unmixing errors, enabling us to resolve multiple EC subpopulations isolated from different tissues in models of chronic tissue injury.

**Research topic(s):**

vascular biology, cell heterogeneity, full spectrum flow cytometry.

## Highlights

• Optimized FSFC panel for in-depth phenotyping of endothelial cell subpopulations.
• Autofluorescence extraction in inflamed or fibrotic tissues.
• Trajectory analysis to track cellular plasticity over time.
• Full spectrum cell sorting to isolate rare endothelial subpopulations.

## Graphical abstract

**Figure:**
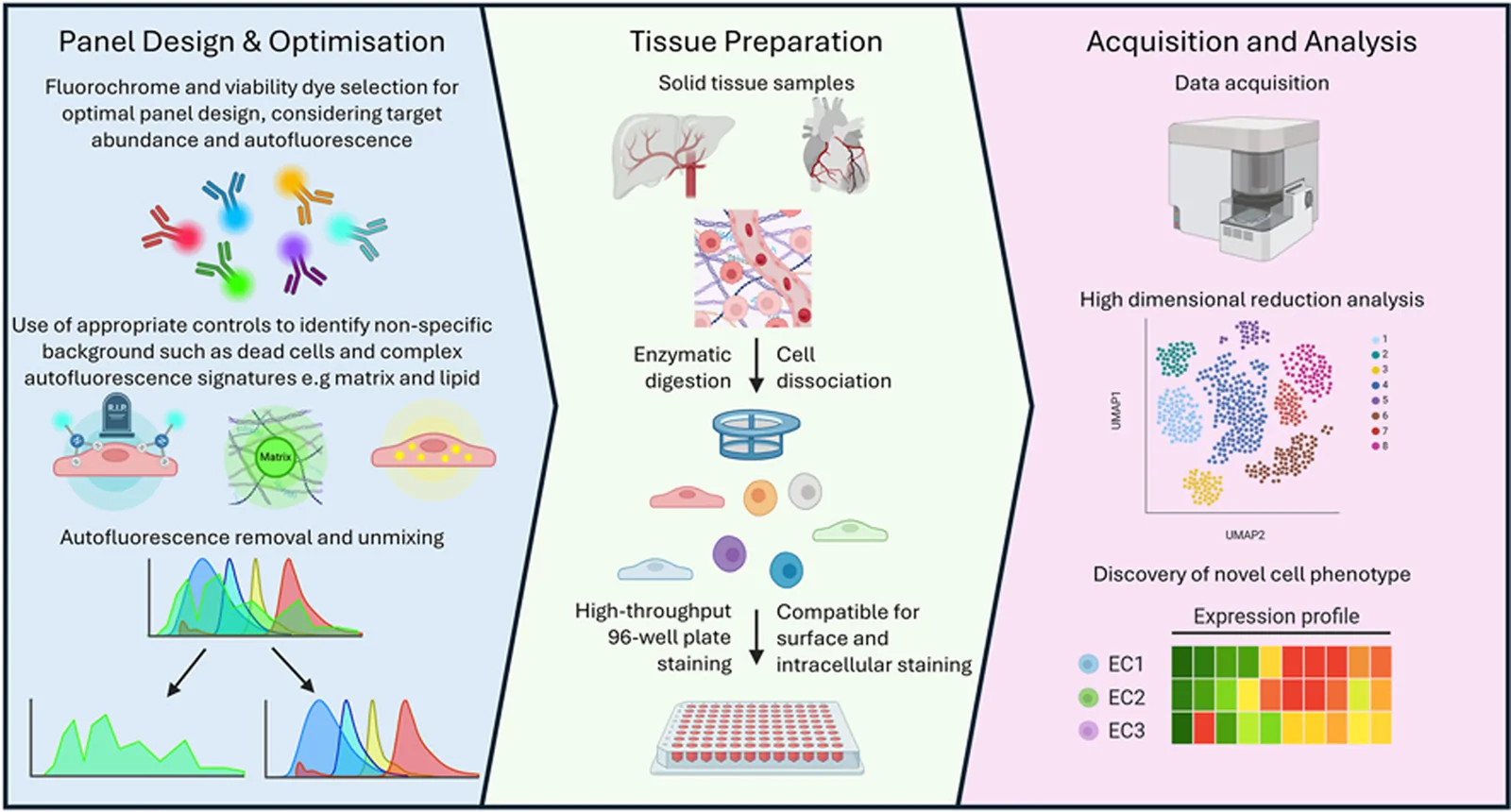

## 1 Introduction

Phenotyping of single cells has provided critical insights into cellular heterogeneity and function with major implications for developing novel targeted therapies. Single-cell RNA sequencing (scRNAseq) has become the gold standard approach for characterising tissue- and disease-specific cellular heterogeneity. However, reliance on transcriptomic data has key limitations: RNA abundance does not always parallel protein expression, and information regarding post-transcriptional or post-translational modifications, as well as cell-surface protein-mediated interactions, are not captured [1].

Full spectrum flow cytometry (FSFC) is an emerging technology that addresses some of these shortcomings by enabling high-dimensional, proteomic, single-cell phenotyping. By measuring protein expression directly, FSFC complements scRNAseq, offering a more comprehensive view of cellular phenotypes. Currently, FSFC is predominantly used for immuno-oncology [2]. Its application to non-immune cell populations and heterogenous solid tissue samples presents additional challenges, as intricate autofluorescence (AF) signatures complicate panel design and spectral unmixing [3].

Endothelial cells (ECs) exemplify a challenging cell population for FSFC. Most studies of EC heterogeneity in health and disease have relied on scRNAseq-based phenotypic assessment [4], [5], [6], while single-cell proteomic analysis remains limited, in part due to their challenging AF signatures [7]. This issue is amplified in steatotic or fibrotic tissues, where excessive lipids and extracellular matrix increase AF [8], [9].

To address these challenges, we developed and optimized a FSFC protocol for phenotyping ECs from murine liver and heart samples. We have combined this FSFC approach with immunofluorescence and scRNAseq analysis to identify the expansion of fibrosis-associated EC (FAEC) subpopulations during models of liver fibrosis [10]. Here, we detail critical considerations, including the selection of an appropriate viability dye, fluorochrome optimisation and strategies for efficient AF extraction, emphasising the importance of tissue-, sex- and model-specific controls to account for different sources of AF. This protocol also describes a downstream analytical pipeline for dimensionality reduction, cluster visualization and trajectory analysis of diverse endothelial subpopulations. Furthermore, we demonstrate the successful isolation of rare EC subpopulations through full spectrum sorting.

This study provides a versatile framework for applying FSFC to diverse solid tissues and non-immune cell types. It also highlights the ability of FSFC to aid the discovery of rare cell populations, describe phenotypic changes and cell plasticity, and identify potential novel therapeutic targets.

## 2 Methods

### 2.1 Animals

C57BL/6 mice at 8-10 weeks old were used for all experiments. Food and water were provided ad libitum. Individually ventilated cages were used in a temperature-controlled facility. A 12-h light: 12-h dark cycle was maintained. All procedures used in this study follow ARRIVE guidelines, were approved by the Animal Use and Care Committee of Queen Mary University of London (QMUL) and were in accordance with national and international regulations (Project license: PP0505769).

### 2.2 Induction of liver injury using CCl_4_ injections

CCl_4_ was diluted 1 in 5 in peanut oil and was administered with an intraperitoneal injection at a dose of 0.8 mg/kg in 100 μl. Injections were administered twice a week for 4 or 8 weeks. No treatment or sham injections of 100 μl peanut oil were used as a control.

### 2.3 Induction of metabolic dysfunction-associated steatohepatitis using a Western diet

To model a Western diet and induce hepatic steatosis and fibrosis, mice were fed the modified Clinton-Cybulsky cholesterol diet up to 24 weeks. The water was also supplemented with carbohydrates (42 g/L) consisting of 55% fructose and 45% sucrose.

### 2.4 Isolation of mouse liver and heart EC

1. Euthanize mice by cervical dislocation and exsanguinate by severing the carotid artery.
  a. Perfuse with sterile deionized phosphate buffered saline (PBS) with 0.5% bovine serum albumin (BSA) and 2 mM EDTA through the left ventricle.
  b. Collect the heart and liver into ice cold PBS.
2. Dissociate tissues in the lab.
  a. Weigh tissues before dissociation.
  b. Mechanically disrupt tissues until homogenized using scissors.
  c. Perform enzymatic digestion using 3 ml pre-warmed (37^°^C) digestion buffer containing 2 mg/ml collagenase, 8U/ml dispase II and 50U/ml DNase dissolved in PBS with CaCl_2_ and MgCl_2_.
  d. Incubate at 37°C for 30 min on a shaker at approximately 180 rotations per minute (rpm).

Note: Tissue weights can be used to calculate cells acquired per gram allowing normalization of cell number between samples.

CRITICAL: The presence of Ca^2+^ and Mg^2+^ ions in digestion buffer is essential for activation of enzymes while DNase reduces cell clumping and therefore reduces the presence of cell doublets in FSFC.

3. Remove undissociated and connective tissue to obtain single cell suspension.
  a. Filter homogenates through 70 μm cell sieves.
  b. Use a 2.5 ml syringe plunger to gently dissociate any remaining tissue through the sieve.
  c. Wash the sample tube with 1 ml Wash Buffer (PBS with 0.5% BSA and 2 mM EDTA) and pass through the sieve.
  d. Wash the sieve with a further 1 ml Wash Buffer.
  e. Centrifuge cell suspensions for 5 min at 400g at 4°C and remove supernatant before resuspending in 1 ml Wash Buffer.
  f. Decant cell suspension through a fresh 70 μm cell sieve and wash through with a further 1 ml Wash Buffer.
  g. Centrifuge cell suspensions for 5 min at 400g at 4°C and remove supernatant before resuspending in appropriate volume of Wash buffer for antibody staining.

Note: We suggest resuspending the cell pellets in 200 μl for profiling EC of the heart and 650 μl for liver EC profiling.

CRITICAL: Choose a resuspension volume that allows for additional controls to be run including unstained and Fluorescence Minus One (FMO) controls.

### 2.5 Flow cytometry staining

1. Transfer 50 μl of cell suspension to each well of a V-bottomed 96-well plate.
  a. Wash with 200 μl PBS by centrifuging the samples at 400g for 5 min.
  b. Resuspend cell pellets in 50 μl Fc blocking solution (diluted 1:500 in PBS).
  c. Incubate plate on ice for 30 min in the dark.
  d. Wash with 200 μl PBS by centrifuging the samples at 400g for 5 min at 4^°^C.
  e. Resuspend pellet in 50 μl PBS containing the LIVE/DEAD Fixable dead cell dye.
  f. Incubate plate on ice for 30 min in the dark.
  g. Wash with 200 μl PBS by centrifuging the samples at 400g for 5 min at 4^°^C.
2. Prepare antibody staining panels ensuring you have the following groups:
  a. Complete panel containing optimized dilutions of each individual antibody diluted in the Brilliant Stain Buffer if required (when containing more than one Brilliant dye).
  b. Fluorescence FMO control samples from which each individual antibody is excluded in turn.
  c. Unstained control samples.

Example: A 5-stain panel containing antibodies A, B, C, D and E would require 7 wells for each sample assessed.

1: A, B, C,D, E (Complete panel)
2: B, C, D, E (FMO for A)
3: A, C, D, E (FMO for B)
4: A, B, D, E (FMO for C)
5: A, B, C, E (FMO for D)
6: A, B, C, D (FMO for E)
7: Antibody diluent only (Unstained control)

Note: Staining performed in 5 ml FACS tubes can allow for higher volume for washes steps; for staining performed in plates, leave an empty well around each sample to avoid cross contamination. Additional washes may be required for complex tissue digests.

3. Antibody panel staining.
  a. Resuspend cells in 50 μl of each antibody panel to individual wells.
  b. Incubate plate on ice for 30 min in the dark.
  c. Wash 2x with 200 μl PBS by centrifuging the samples at 400g for 5 min at 4^°^C.
4. (Optional) Intracellular Staining. (Required for TAGLN staining in our panel).
  a. Resuspend cells in 50 μl the intracellular fixation and permeabilisation buffer.
  b. Incubate plate at room temperature for 20 min in the dark.
  c. Wash with 200 μl permeabilisation wash buffer by centrifuging the samples at 400g for 5 min.
  d. Add 50 μl of each antibody dilution to individual wells (diluted in permeabilisation wash buffer).
  e. Incubate plate at room temperature for 30 min in the dark.
  f. Wash ×2 with 200 μl permeabilisation wash buffer by centrifuging the samples at 400g for 5 min.
  g. Resuspend the samples in 300 μl MACS buffer (PBS with 2% FBS, 2 mM EDTA).
  h. Filter and transfer samples to FACS tubes.

### 2.6 Protocol for single stain reference controls

The staining protocol described above can be used to create single cell reference controls using cells.

For staining of compensation beads to be used as single cell reference controls details are below.

a. Vortex beads.
b. Add 20 μl beads in each well along with 80 μl PBS (beads quantity and volume can be adjusted based on beads' manufacturer protocol).
c. Add 1 μl antibody per well (antibody amount based on titration).
d. Incubate plate at 4^°^C for 30 min in the dark.
e. Wash with 200 μl MACS buffer by centrifuging the samples at 400g for 5 min.
f. Remove the supernatant.
g. Proceed with the Fix/Perm step for intracellular staining markers when required.
h. Resuspend beads in 200 μl MACS buffer.
i. Transfer samples to FACS tubes.

CRITICAL: When setting up these controls, it is important to initially stain both cells and beads to determine which provides the most accurate signal profile for each fluorochrome. Beads often offer a clear, fluorochrome-specific signature that does not rely on cellular expression, which is particularly useful when antibodies target low-abundance markers or rare populations. However, in some cases, the spectral profile of a fluorochrome bound to beads does not fully match its profile when bound to cells, potentially leading to spectral unmixing errors. Therefore, testing both options is essential to ensure optimal unmixing accuracy.

### 2.7 Protocol for unmixing using multi-AF extraction

After sample acquisition, unmixing using multi-AF extraction was performed. The AF Explorer tool in the SpectroFlo® software was used on the unstained sample of each experimental group. To extract multiple AF signatures, the following steps should be followed.

1. Remove doublets and debris from the analysis (Fig. 1A).

**Fig. 1:**
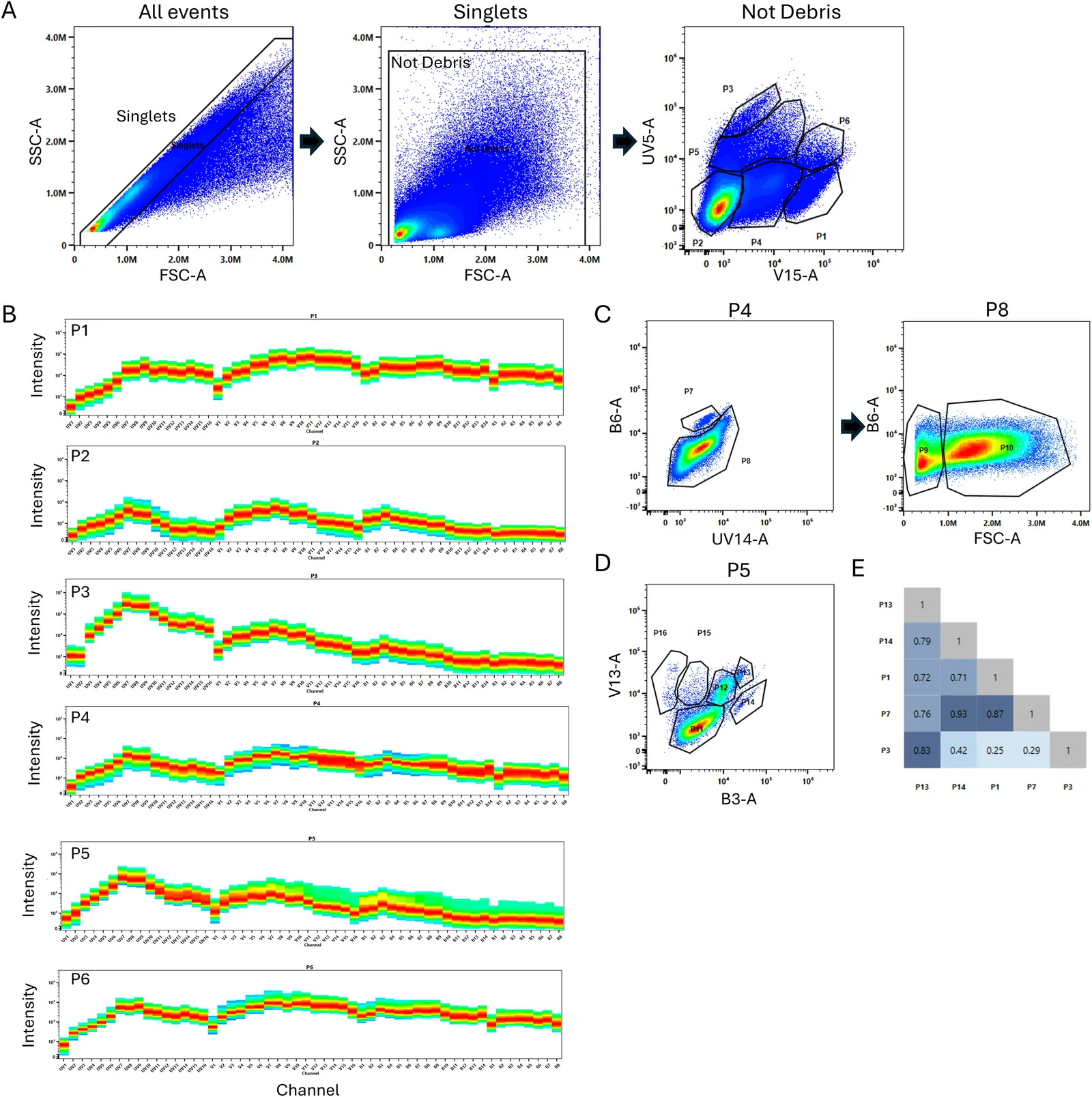
**Description of the workflow for multi-AF extraction.** (A) Gating for the removal of doublets and debris and the identification of multiple AF signatures. (B) Spectral signatures of the 6 AF populations identified with arrows denoting regions of high heterogeneity in spectrum. (C) Gating into P4 to identify populations P7 and P8 and then further gating into population P8 to identify populations P9 and P10. (D) Gating into population P5 to identify subpopulations P11-P16. (E) Similarity matrix for the 5 AF signatures used for unmixing after vetting the AF signatures identified using a cutoff of similarity <0.93.
2. Explore the AF populations of the sample using different fluorescence detectors on the X and Y axes of a density plot.
3. To explore the AF, choose the detectors that reveal the widest variety of AF populations on the spectrum. Note that these might not correspond to the highest intensity of AF.
4. Draw a gate around each AF population (Fig. 1A).
5. Examine the spectral signature of each AF population to ensure that it does not contain more than one AF population. Areas of high spread indicate that that particular gate contains more than one AF population and therefore, further gating is required to isolate the individual AF populations.

Example: By examining the spectral signatures in Fig. 1B, it can be observed that gates P4 and P5 contain more than one AF population each. Therefore, further gating was performed within gates P4 (Fig. 1C) and P5 (Fig. 1D). This led to the identification of 3 populations within gate P4 and 6 populations within gate P5.

6. Extract the AF of each population.
7. Vet the identified AF using an appropriate similarity threshold (≤0.98).

Note: The presence of multiple AF populations with similar signatures can increase the panel complexity, resulting in unmixing errors. An appropriate similarity threshold should therefore be determined on a case-by-case basis. For this example, a similarity threshold of ≤ 0.93 was used, which resulted in the identification of 5 unique AF populations (Fig. 1E).

8. Perform unmixing with multi-AF extraction using the AF signatures identified.

### 2.8 Protocol for computational analysis

Several software platforms support this type of analysis; however, for simplicity, we will describe our pipeline optimized within OMIQ® (Fig. 2).

1. Data pre-processing
  a. Clean up the dataset using the flowAI quality control (QC) algorithm to remove low-quality events [11].
  b. Create a new gate that contains only good quality datapoints. This gate will be used for all downstream analysis.
  c. Scale the data as appropriate to achieve good data visualization.
  d. Apply manual compensation if necessary. Note that compensation can be applied to both fluorescence and AF channels.
  e. Gate the cell populations to identify the cell population of interest that will be used for dimensionality reduction analysis.
2. Dimensionality reduction
  a. Perform unsupervised clustering using the FlowSOM algorithm [12].
    i. Choose the markers to be used for clustering based on the biological question at hand.
    ii. Determine the number of meta-clusters using an appropriate K value.
    iii. Once the clusters have been generated, create a gate for each cluster to enable cluster inspection and visualization.
  b. Create a Uniform Manifold Approximation and Projection (UMAP) plot to visualize the clusters [13].
    i. Choose the markers to be used for the visualization (same as the ones used for clustering).
    ii. Input the number of nearest neighbours and minimum distance to be used for UMAP generation. Note that the number of nearest neighbours indicates the number of nearest datapoints the algorithm uses when mapping local connections. The minimum distance controls how closely the datapoints are positioned in the low-dimensional map.
3. Post-processing analysis
  a. Manually merge clusters if necessary, using Boolean filters. (e.g Clusters that positively expressed identical EC markers above FMO Median Fluorescence Intensity (MFI) cut-off were considered to have the same phenotypic signature and therefore merged).
  b. Use the Wishbone algorithm [14] to perform trajectory analysis.
    i. Create a diffusion map to generate the vectors that will be used for the trajectory analysis.
    ii. Create a gate for the starting cell of the trajectory.
    iii. Perform trajectory analysis using the starting cell gate and the appropriate vectors generated using the diffusion map.

**Fig. 2:**
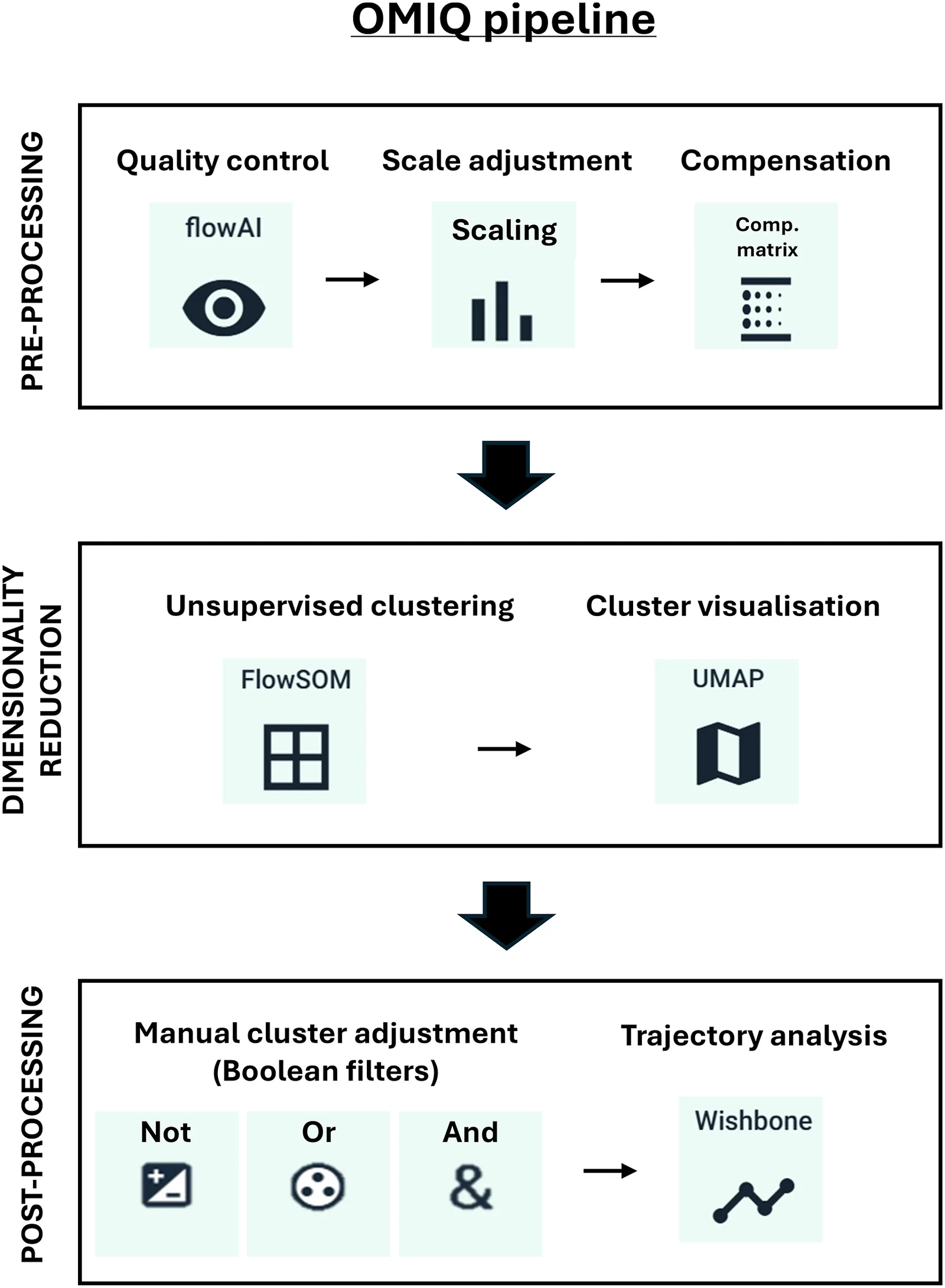
**Outline of the OMIQ pipeline used for high dimensional analysis of full spectrum flow cytometry data.** For data pre-processing, the FlowAI algorithm was used for quality control, followed by data scaling and manual compensation as required. Unsupervised clustering was then performed using the FlowSOM algorithm, while cluster visualization was achieved through UMAP plots. During post-processing, clusters were manually merged or split as deemed necessary, and trajectory analysis was conducted using the Wishbone algorithm.

A complete summary of all experimental and computational steps carried out in this study is presented in Fig. 3 with a list of all reagents provided in Table 1.

**Fig. 3:**
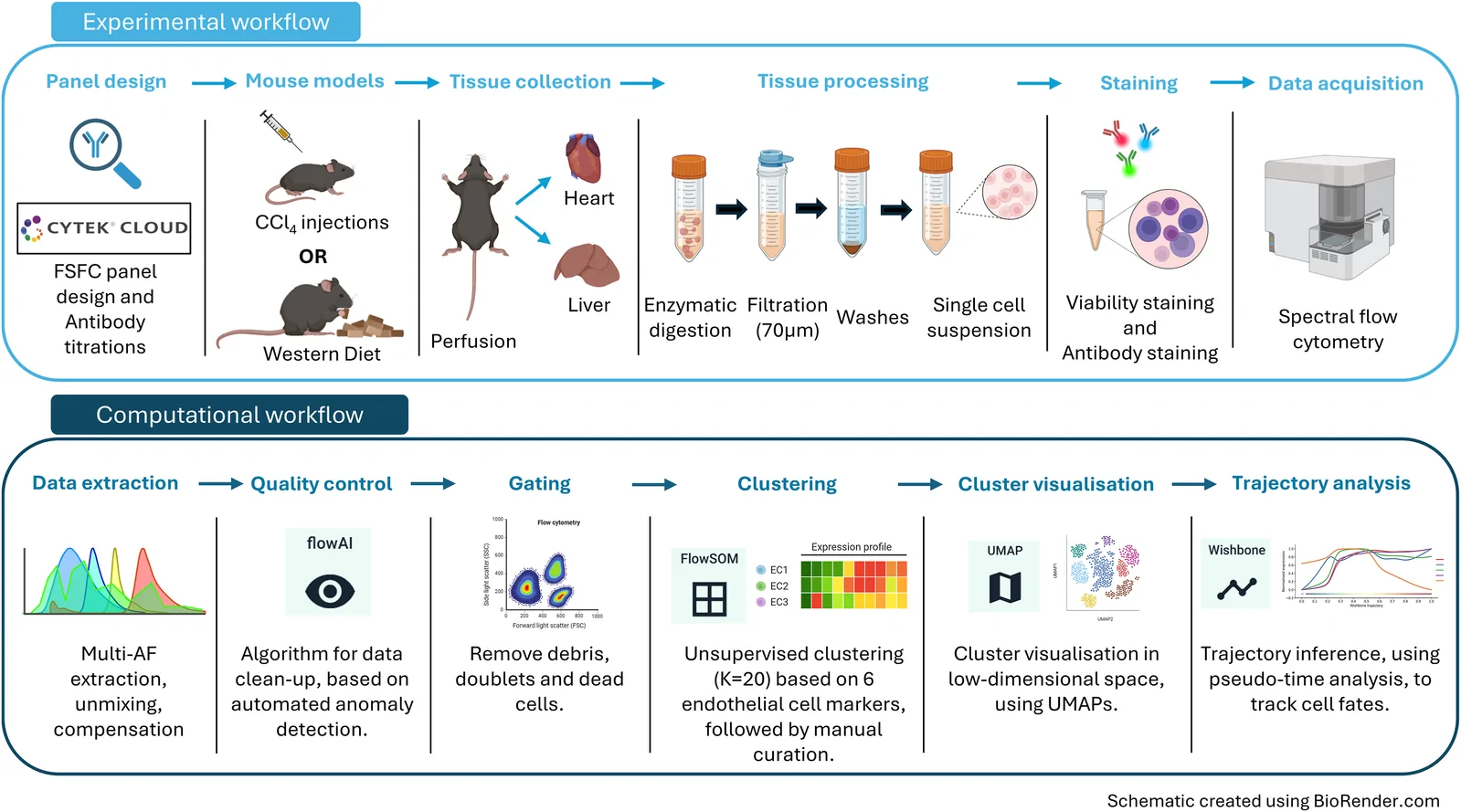
**Schematic summary of the individual experimental and computational workflows detailed in the methodology section.** Created with BioRender.com.

**Table 1:** Key resources table.

REAGENT or RESOURCE	SOURCE	IDENTIFIER
Antibodies		
CD31 (Clone 390) BV785	Biolegend	102435
CD45 (Clone 30-F11) BUV395	BD Bioscience	565613
Thrombomodulin (Clone LS17-9) PE	Fisher Scientific	12-1411-82
EMCN (Clone V.7C7) eFluor660	Fisher Scientific	50-5851-82
LYVE1 (Clone ALY7) PE-Cy5.5	Novus	NBP1-43411
PDPN (Clone 8.1.1) PE-Cy7	Biolegend	127411
MCAM (Clone ME-9F1) BV650	Biolegend	740636
LY6A (Clone D7) BV480	Biolegend	746663
ICAM1 (Clone YN1/1.7.4) PerCP-Cy5.5	Biolegend	116123
TAGLN (Clone TAGLN/247) AF488	Novus	NBP2-47688AF488
THY1.2 (Clone 30-H12) AF700	Biolegend	105320
PDGFRa (Clone APA5) APC-eFluor780	Fisher Scientific	47-1401-82
CD34 (Clone MEC14.7) BV421	Biolegend	119321
		
Chemicals, peptides, and recombinant proteins		
Phosphate Buffered Saline (PBS)	Fisher Scientific	BR0014G
Bovine Serum Albumin (BSA)	Fisher Scientific	BP9702-100
EthyleneDiamineTetraacetic Acid (EDTA)	Sigma Aldrich	E7889-100 ML
Collagenase	Gibco	17018-029
Dispase II	Roche	04942078001
DNase	Invitrogen	18047-019
PBS with CaCl_2_ and MgCl_2_	Gibco	14170-088
70 μm cell sieve	Scientific Laboratory Supplies	431751
Fc blocking solution	Miltenyi Biotec	130-092-575
Live/Dead™ Green Fixable dye	Invitrogen	L23105
Brilliant stain buffer	BD Biosciences	554656
Intracellular fixation and permeabilisation buffer	Invitrogen	00-5521-00
Permeabilisation wash buffer	Invitrogen	00-8333-56
Foetal Bovine Serum (FBS)	Gibco	A4766801
UltraComp eBeads™ Compensation Beads	Fisher Scientific	01-2222-41
		
Experimental models: Organisms/strains		
Carbon Tetrachloride CCl_4_	Sigma-Aldrich	289116-100 ML
Modified Clinton-Cybulsky cholesterol diet	IPS Product Supplies	58R6
Fructose	Sigma-Aldrich	F0127-1 KG
Sucrose	Sigma-Aldrich	S9378-1 KG
Peanut oil	Sigma	P2144-250 ML
C57/BL6 Mice	Charles Rivers Labroatories	
Software and algorithms		
SpectroFlo® 3.3.0 software	Cytek®	
Cytek® Cloud	Cytek®	
OMIQ®	Dotmatics	
		
Other		
4 laser Aurora (UV, V, B, R) spectral flow cytometer	Cytek®	

## 3 Results

### 3.1 Panel design

When determining the size and spectral range of a panel, it is important to consider the capabilities of the equipment, such as the number of lasers and detectors available, which will dictate the range of fluorochromes that are compatible. Availability of more detectors can lead to lower panel complexity scores even for the exact same fluorochromes, thereby reducing unmixing errors. The panel we designed was for the 4 laser Cytek® Aurora (UV/V/B/R, 54 + 3 channel) platform. To calculate the fluorochrome similarity and the complexity index of the panel, the Spectral Viewer tool (available through the Cytek® Cloud) can be used. We show the similarity score for the 14 fluorochromes in our panel with similarity ranging from 0 to 0.94 (Fig. 4A); Cytek® recommends that similarity can be tolerated up to 0.98 within a panel. The complexity of the panel is a measure of the spectral uniqueness of its fluorochromes, with lower values predictive of a simpler unmixing process. The stain reduction index matrix (Fig. 4B) is used to quantify how the stain index of each fluorochrome is reduced due to the presence of the other fluorochromes within the panel. Finally, the spillover matrix (Fig. 4C) identifies potential spillover of the emission wavelengths of each fluorochrome into other channels. Together these tools act as a guide for the selection of fluorochromes with unique signatures as well as highlight potential incompatible fluorochrome combinations. However, all combinations require experimental optimisation including antibody titrations and assessment of the impact of complex autofluorescence signatures to minimize unmixing errors which we will address in more detail.

**Fig. 4:**
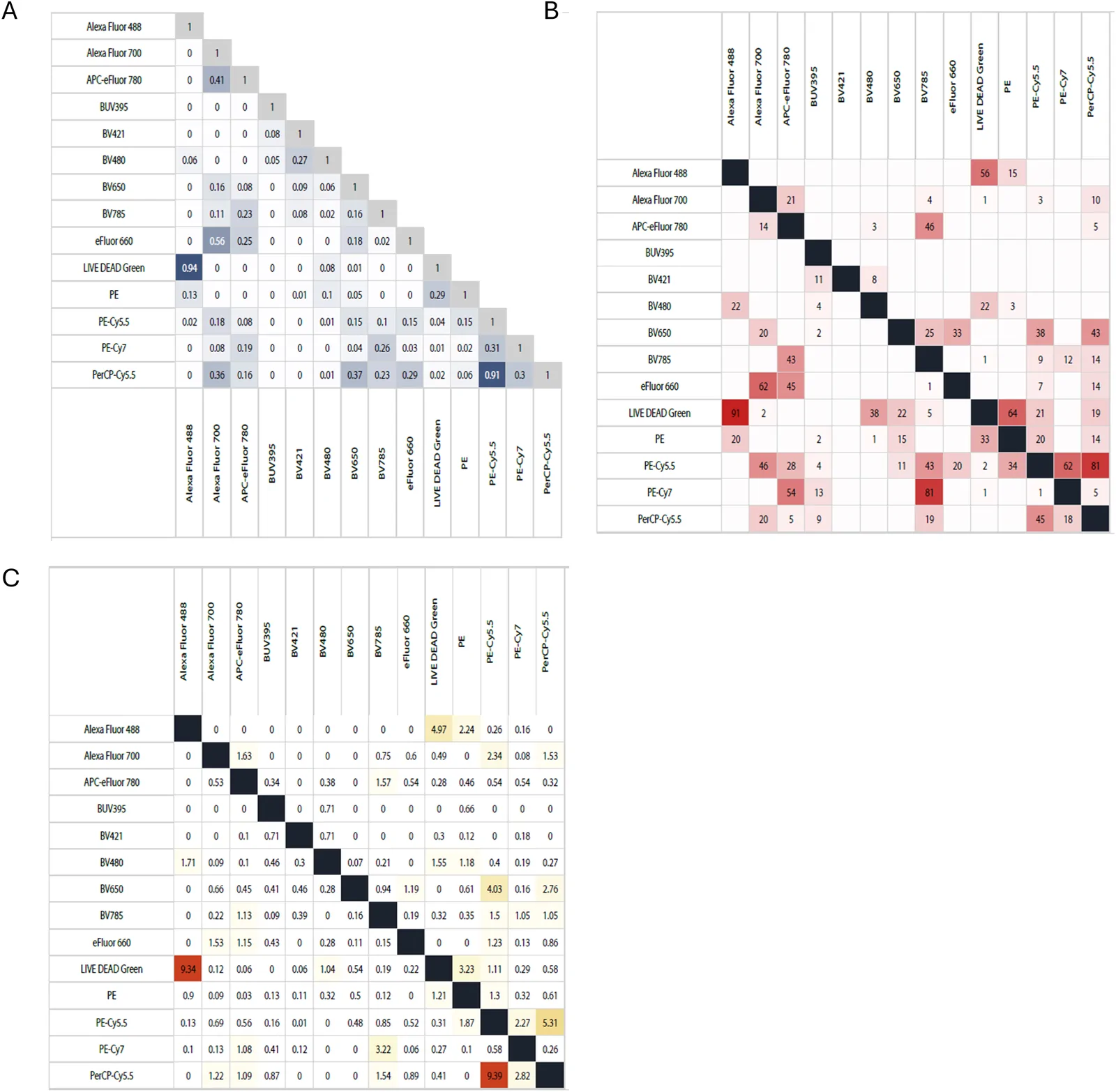
**Properties of the 14-colour endothelial cell panel.** The 14 fluorochromes used in the endothelial cell antibody panel were assessed using the Cytek® Spectral Viewer tool to generate a (A) complexity index, (B) stain reduction index and (C) spillover spread matrix for the panel.

To identify specific cell types within whole tissue samples it is useful to define inclusion and exclusion markers to enhance the analysis of a chosen population. To build a comprehensive EC panel, we consulted reports that used several different experimental approaches to identify and study different EC subpopulations. These approaches included lineage tracing studies [15], [16], transcriptomic studies [17], [18] and EC assessment by immunofluorescence (IF) [10], [19], [20]. Our own assessment using IF allowed us to visualize the selectivity of different markers for specific EC subpopulations, exemplified in Fig. 5 by the expression of thrombomodulin (TM) and endomucin (EMCN). Both markers are EC-specific and highly zonated. Within the hepatic vasculature (Fig. 5A), low expression can be observed in peri-portal (PP) regions, while high expression is noted around peri-central (PC; Zone 3) (Fig. 5B; explored in more detail in Gkantsinikoudi et al.*,* 2026 [10]). In the heart, the same markers demonstrate ventricular zonation (Fig. 5C), displaying higher expression in endocardial EC and epicardial regions with lower expression in microvascular EC of the mid-ventricular regions (Fig. 5D). Based on this approach, 6 EC identity markers were chosen for this FSFC panel due to their EC-restricted expression profiles and their ability to identify different subpopulations within both the liver and heart tissue. These EC markers were combined with 3 markers of EC activation (ICAM1, LY6A and CD34) and 3 markers of mesenchymal transition (transgelin (TAGLN), THY1.2, and platelet-derived growth factor receptor α (PDGFRα)) allowing us to study the dynamic changes in EC phenotypes and induction of endothelial-to-mesenchymal transition (EndMT) in models of fibrosis [10]. A detailed description of the markers chosen for this panel and their function in the liver and heart is presented in Table 2. The pan-leukocyte marker CD45 was included in our panel to exclude a small proportion of CD31^+^ immune cells, while a viability dye was also used to exclude dying or dead cells from the analysis. The final gating strategy used to identify the total population of endothelial cells is presented in Fig. 6A–D.

**Fig. 5:**
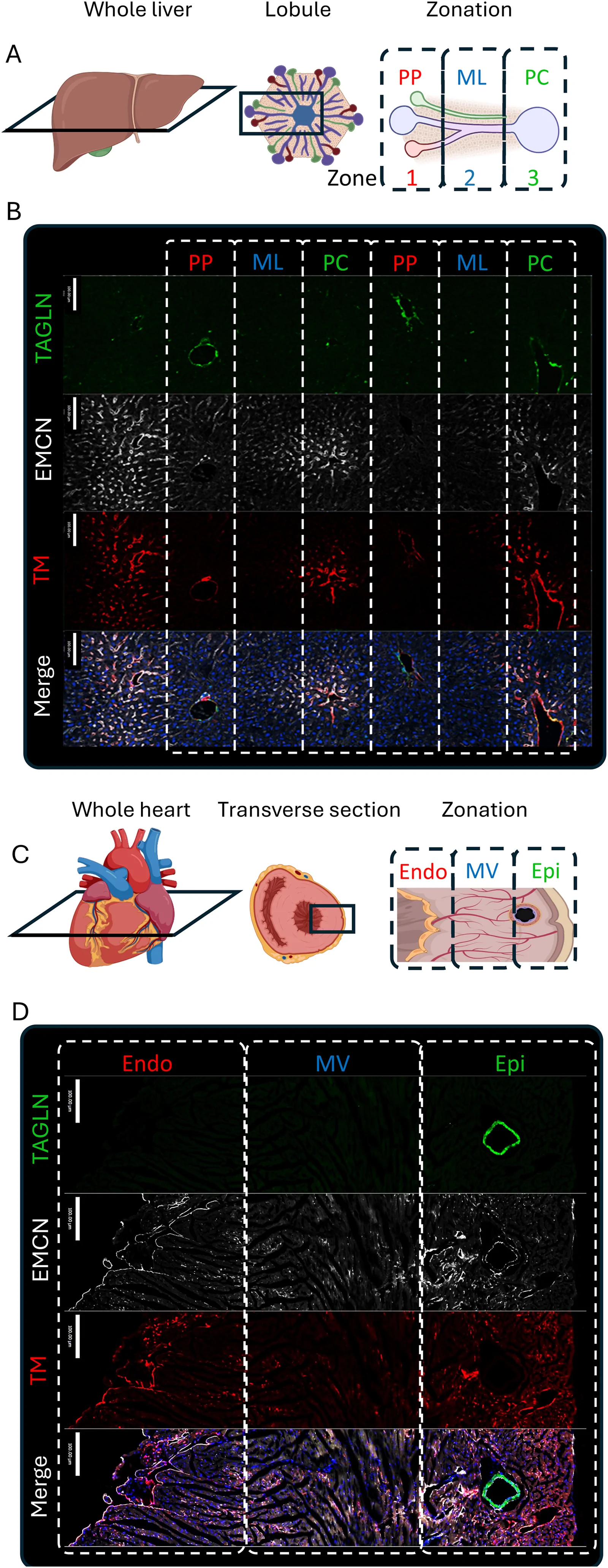
**Zonated expression of endothelial markers in the liver and heart.** (A) Schematic of a liver cross section. The liver is organized into hexagonal lobules that consist of three distinct zones, termed peri-portal (PP; Zone 1), mid-lobular (ML; Zone 2) and peri-central (PC; Zone 3) zones. (B) Representative confocal image of a liver section stained for transgelin (TAGLN, green; smooth muscle cells/mesenchymal cells), endomucin (EMCN, white; endothelial cells), thrombomodulin (TM, red; endothelial cells) and Hoechst (blue; nuclei). Scale bars = 100 μm. (C) Schematic of a transverse cross section of the heart. The left ventricle is presented from the endocardium (Endo; left) through the mid-ventricle (MV) to the epicardium (Epi; right). (D) Representative confocal image of a heart section stained for transgelin (TAGLN, green; smooth muscle cells surrounding the coronary artery), endomucin (EMCN, white; endothelial cells), thrombomodulin (TM, red; endothelial cells) and Hoechst (blue; nuclei). Scale bars = 100 μm. Schematics created with BioRender.com.

**Table 2:** Description of markers included in the FSFC endothelial panel.

Marker	Description
CD31	Highly expressed in all endothelial cells [21].
CD34	Marker of endothelial progenitors, expression reported in EndMT cells [22].
CD45	Immune cell marker used for the exclusion of immune cells.
EMCN	Endothelial marker that regulates the adhesion of leukocytes. It is mainly found in venous endothelium and capillaries. In the liver, its expression is zonated and marks zone 3, peri-central endothelial cells [21]. [23]
ICAM1	Inflammatory marker, facilitates the adhesion and migration of leukocytes [24].
LY6A (also known as Sca-1)	Marker of liver sinusoidal endothelial cells in liver disease. In the heart, LY6A^+^ EC contribute to the microvascular capillary network [25]. [26]
LYVE1	Expressed on liver sinusoidal endothelial cells and lymphatic vessels. Marks zone 2, mid-lobular endothelial cells in the liver. In the heart, in addition to lymphatic vessels, LYVE1 is expressed by a subset of endocardial endothelial cells [21].
MCAM (also known as CD146)	Endothelial marker. Increased during fibrosis and in cells undergoing EndMT [27].
PDGFRα	Expressed on endothelial cells undergoing EndMT [28].
PDPN	Marker of lymphatic endothelial cells [21].
TAGLN	Expressed on endothelial cells undergoing EndMT [29].
THY1.2 (also known as CD90)	Expressed on endothelial cells undergoing EndMT [30].
TM	Endothelial marker, with anti-coagulant function [21].

**Fig. 6:**
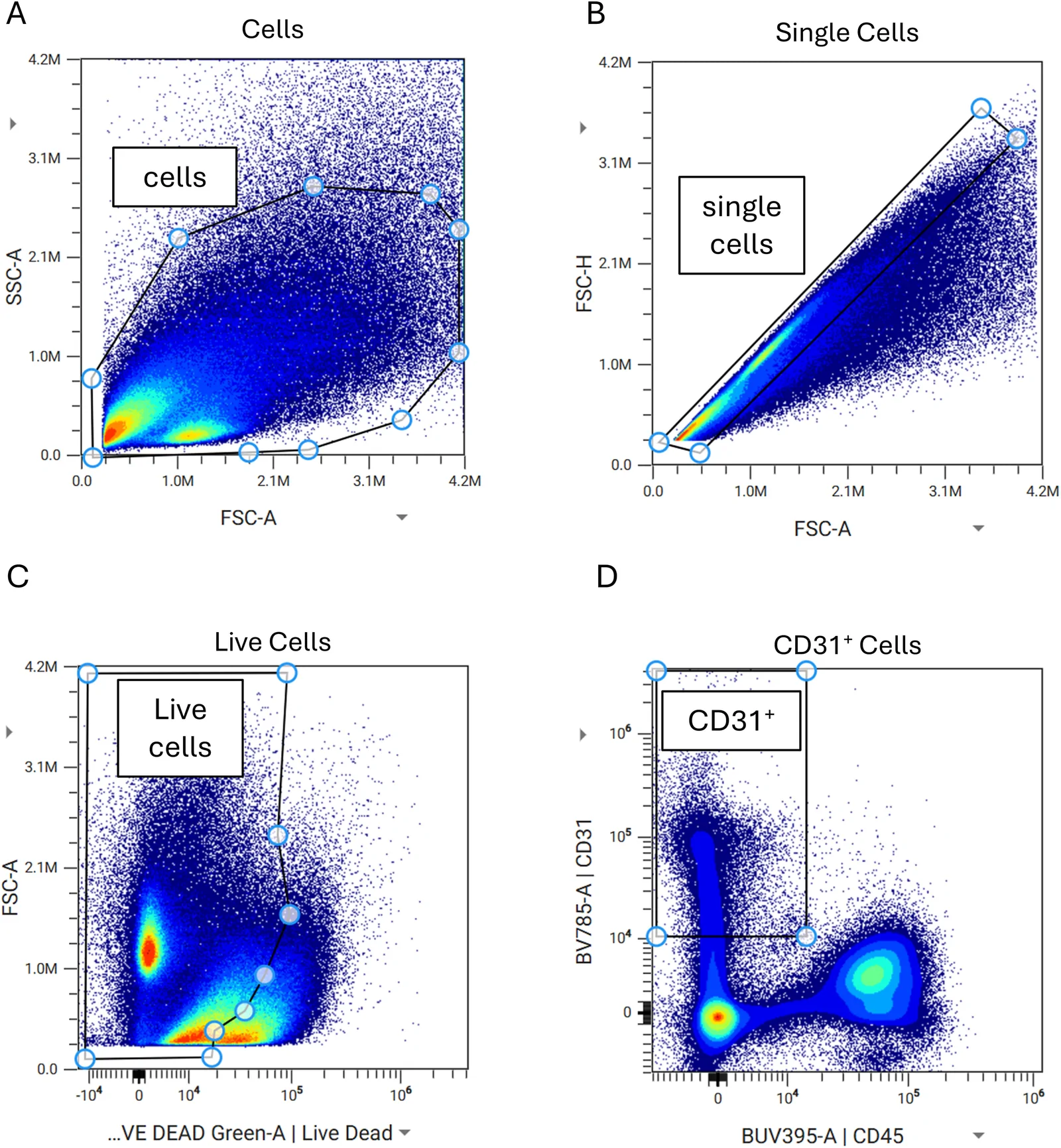
**Gating strategy for the identification of endothelial cells.** (A) The total cell population was identified using the FSC-A vs SSC-A plot. (B) Doublets were excluded based on the FSC-A vs FSC-H plot. (C) Live cells were selected using the LIVE/DEAD Green Fixable viability dye. (D) The total population of endothelial cells was selected based on positive signal for CD31 and negative signal for CD45 (CD31^+^CD45^−^ cells). All EC subpopulations were identified in CD31^+^CD45^−^ gates.

### 3.2 Optimisation of the viability dye and antibody dilutions

#### 3.2.1 Viability dye selection

The selection of a suitable viability dye is the crucial first step in optimizing a successful panel. Two of the most common and spectrally diverse types of viability dyes are DNA-binding (e.g. DAPI or Hoechst) and amine-reactive dyes (e.g. LIVE/DEAD Fixable Viability Dyes (Invitrogen), Zombie Fixable Viability Dyes (Biolegend)). We found that using a DNA-binding dye was not the most suitable approach for our panel: due to the fixation and permeabilisation step of our protocol, DNA staining of live cells was still evident, even after optimisation efforts. Therefore, we assessed the staining profiles of three LIVE/DEAD Fixable Dye reagents to identify a potentially compatible fluorochrome to include in the panel. There are striking differences in the cellular distribution profiles between the three fluorochromes tested (Fig. 7A–C) with Live/Dead Green chosen for the clearest segregation of viable cells. This finding was contrary to our initial expectation that NIR viability dye would be the most suitable choice because its emission wavelength lies within a low-AF zone of the spectrum. However, one of the chemical compounds used to generate the liver injury mouse model resulted in high AF in that region. This highlights the importance of considering model-specific sources of AF when designing a FSFC panel.

**Fig. 7:**
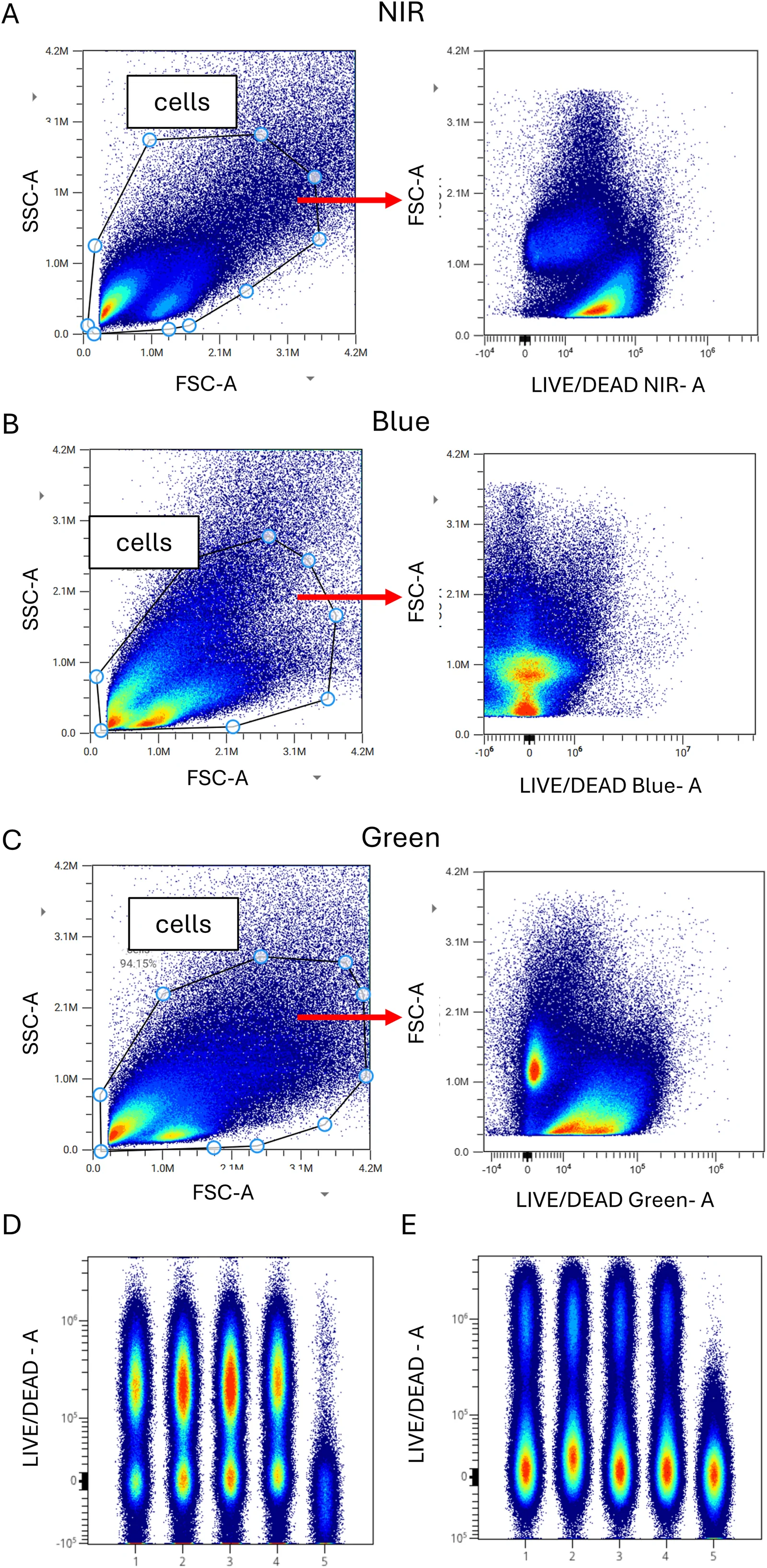
**Optimisation of LIVE/DEAD Fixable dead cell staining.** (A-C) Scatter plots of liver cell suspensions stained with (A) LIVE/DEAD NIR, (B) LIVE/DEAD Blue and (C) LIVE/DEAD Green Fixable dead cell dyes. (D, E) Titrations of the LIVE/DEAD Fixable dead cell dye on (E) heat killed and (F) non-heat killed cells from whole liver single cell suspensions. The dilutions used were (1) 1: 1,000, (2) 1: 2,000, (3) 1: 5,000, (4) 1: 10, 000 and (5) unstained.

For the viability dye titration experiments, heat-killed (90^°^C for 5 min; Fig. 7D) and non-heat killed samples (Fig. 7E) were stained separately. Before data acquisition, stained and unstained cells were mixed in the same tube, to obtain a clear positive and negative peak for each sample. The 1:5000 dilution was the one with the highest Stain Index, providing the clearest resolution between live and dead cells with minimal signal distortion.

#### 3.2.2 Antibody selection

There are several factors that can influence the selection of conjugated antibodies for FSFC panels. It is essential to understand the properties of the target cells, particularly the expression levels of the chosen antigens and whether they are co-expressed on the same cell populations. Whenever possible, avoid combining antibodies that bind to epitopes located in close physical proximity, as this can impair antibody affinity through steric hindrance or stoichiometric inhibition.

When selecting fluorochromes, several considerations are important. Fluorochromes with significant spectral overlap or those contributing to unmixing-dependant spreading due to spillover, instrument noise and statistical variation, should ideally be assigned to antigens that are not co-expressed on the same cell. In addition, the gating strategy and co-expression of markers of interest should be considered when selecting fluorochromes. This approach helps to minimize data spread and improve the accuracy of spectral unmixing. Moreover, bright fluorochromes should be matched with antigens with unknown distribution and low expression levels, while dimmer fluorochromes are better suited for highly abundant targets with well-defined expression patterns. Using dim fluorochromes for low-abundance proteins can results in signal loss due to high background and AF. Similarly, assigning bright fluorochromes to highly expressed proteins may lead to excessive signal spread, which can compromise resolution and cause inaccurate spectral unmixing. For example, in our panel, we assigned the highly expressed CD45 to the lower-brightness BUV395 for a negative gate, whereas the CD31 was paired with the brighter BV785 to maximise detection sensitivity. Similarly, for the core endothelial markers, bright fluorochromes with minimal spectral similarity to CD31 and to one another were selected, including PE for TM, eFluor660 for EMCN, PE-Cy5.5 for LYVE1, PE-Cy7 for PDPN and BV650 for MCAM.

Availability of antibody clones and fluorochrome conjugates is also a major consideration. For non-immune cell markers, commercial demand is lower, which often limits the range of available conjugates and can constrain panel design. For example, at the time of panel design, the available options for LYVE1 and ICAM1 were limited, resulting in the inclusion of both PE-Cy5.5 and PerCP-Cy5.5, two fluorochromes with high spectral similarity. Their inclusion within the same panel was considered acceptable based on Cytek recommendations for fluorochromes with <98% similarity and the overall panel remained low in complexity (complexity score 8.02). Thus, while greater fluorochrome separation would be desirable, optimal fluorochrome selection is not always feasible due to limitations in commercially available antibody-fluorochrome combinations. To determine the suitability of selected clones and fluorochromes for FSFC applications, we utilized databases such as CiteAb and Biocompare.

Titration of the selected antibodies (Table 1) was performed by incubation of individual antibodies over a dilution range from 1:20 - 1:640 in buffer containing 1:5000 dilution of the viability dye. The optimal dilution for each antibody was then determined by assessing the separation of the positive and negative cell populations, with minimal background signal (Stain Index). For this assessment, only the live cells were taken into consideration, as dead cells can non-specifically bind to antibodies leading to high background and false positive signal. Finally, all dilutions were validated as part of a complete panel. The plots for all antibody titrations of this EC panel are presented in Fig. 8A–M, with red boxes highlighting the selected dilutions for each antibody. The stain index of all antibody dilutions assessed is presented in Fig. 8N. In some cases where dilutions displayed similar stain index, the lowest antibody concentration was chosen to reduce the economic burden of the panel design.

**Fig. 8:**
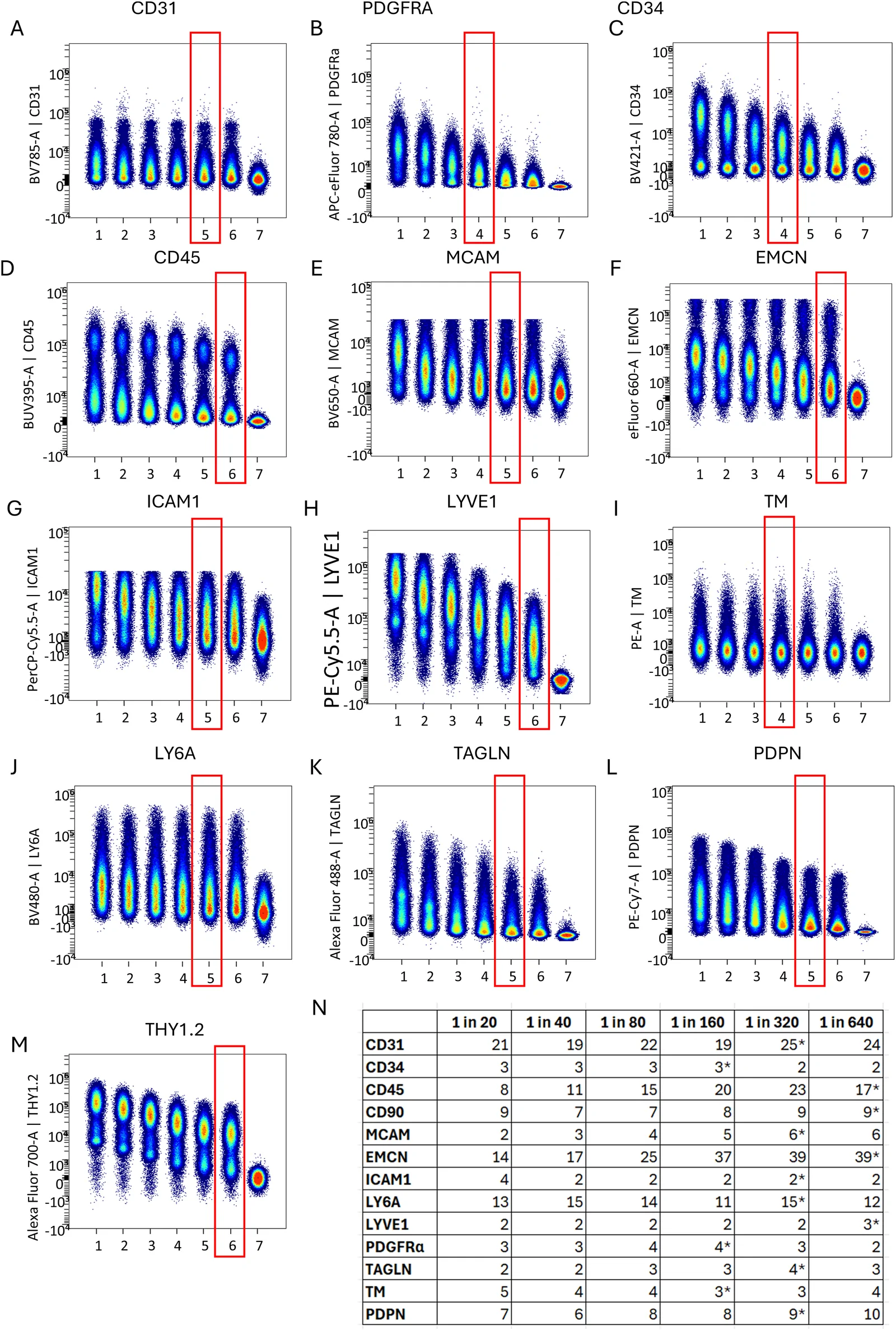
**Titration of all endothelial cell panel antibodies.** Each antibody was diluted at (1) 1:20, (2) 1:40, (3) 1:80, (4) 1:160, (5) 1:320, (6) 1:640, while an unstained sample (7) was also used for comparison. The antibodies tested included (A) CD31, (B) platelet-derived growth factor receptor α (PDGFRα), (C) CD34, (D) CD45, (E) melanoma cell adhesion molecule (MCAM), (F) endomucin (EMCN), (G) ICAM1, (H) LYVE1, (I) thrombomodulin (TM), (J) LY6A, (K) transgelin (TAGLN), (L) podoplanin (PDPN) and (M) THY1.2. Red boxes indicate the final dilution chosen for each marker. (N) Table of stain index calculations for each antibody dilution used. Asterisks indicate the chosen dilution.

### 3.3 Unmixing controls and extraction of complex autofluorescence signatures

The next step was to generate a library of single stain reference controls for all fluorochromes, as these represent an essential tool for correct data unmixing. Reference controls capture the unique spectral signature of each fluorochrome, enabling the software to correctly assign fluorescence signals to each fluorochrome across the spectrum. They should initially be tested on both beads and cells to determine the most accurate signal for each fluorochrome. For tandem dyes, the reference control should be prepared using the exact same antibody vial to minimize lot-to-lot variation. For non-tandem dyes, a different antibody may be used if necessary to achieve a strong positive signal and correct brightness. Examples of reference signatures for each fluorochrome can be found on the Cytek® cloud and in the Cytek® Aurora Fluorochrome Selection Guidelines. Additionally, the positive and negative signal for each reference control must originate from the same cell or bead population to ensure consistency. Beyond spectral signature accuracy, signal intensity is also critical: the reference control signal should be at least as bright as the fully stained sample; otherwise, unmixing errors will occur.

The unstained control is one of the most important and critical samples in spectral flow cytometry. It must consist of the exact same cell types as the fully stained samples and include sufficient cell numbers to represent all relevant subpopulations. This ensures accurate AF signature extraction for each sample.

Solid tissue samples present additional complexity, as they contain a variety of different cell types, metabolites, extracellular matrix proteins and connective tissue. These factors contribute to unique AF signatures that vary with tissue composition and cellular complexity. Moreover, AF can be further influenced by chemicals, drugs, treatments, tissue injury, inflammation and fibrosis. Therefore, the most effective strategy for AF extraction in complex tissue and injury models is to capture all the different AF signatures to account for these variations. We used the ‘multi-AF extraction’ tool available through the SpectroFlo® software to identify and extract several AF signatures. It is crucial to include appropriate unstained control samples for all relevant variables, such as time-points, sex and injury models, to avoid potential unmixing errors caused by unique AF signals (Fig. 9A–D). With this approach we identified 4 AF signatures in the healthy heart and up to 6 in the WD hearts, while the WD livers contained up to 9 unique AF signatures. It is important to note that some of the AF signatures are found ubiquitously across all subpopulations (e.g AF1 in the liver) while others are specific for certain cell clusters (e.g AF4). These distinctive features are apparent in both liver and heart (Fig. 9E and F respectively), however we are currently unable to determine the biological implications of these findings. To further highlight the importance of multi-AF extraction from solid tissues in complex injury models, we have provided the NxN plots of a sample to exemplify the differences between unmixing without AF extraction and compensation (Fig. S1), unmixing with total AF extraction and compensation (Fig. S2) and with multi-AF extraction and compensation (Fig. S3) conducted in the same sample. These data show that the application of both multi-AF and manual compensation is the most effective approach to reducing unmixing errors.

**Fig. 9:**
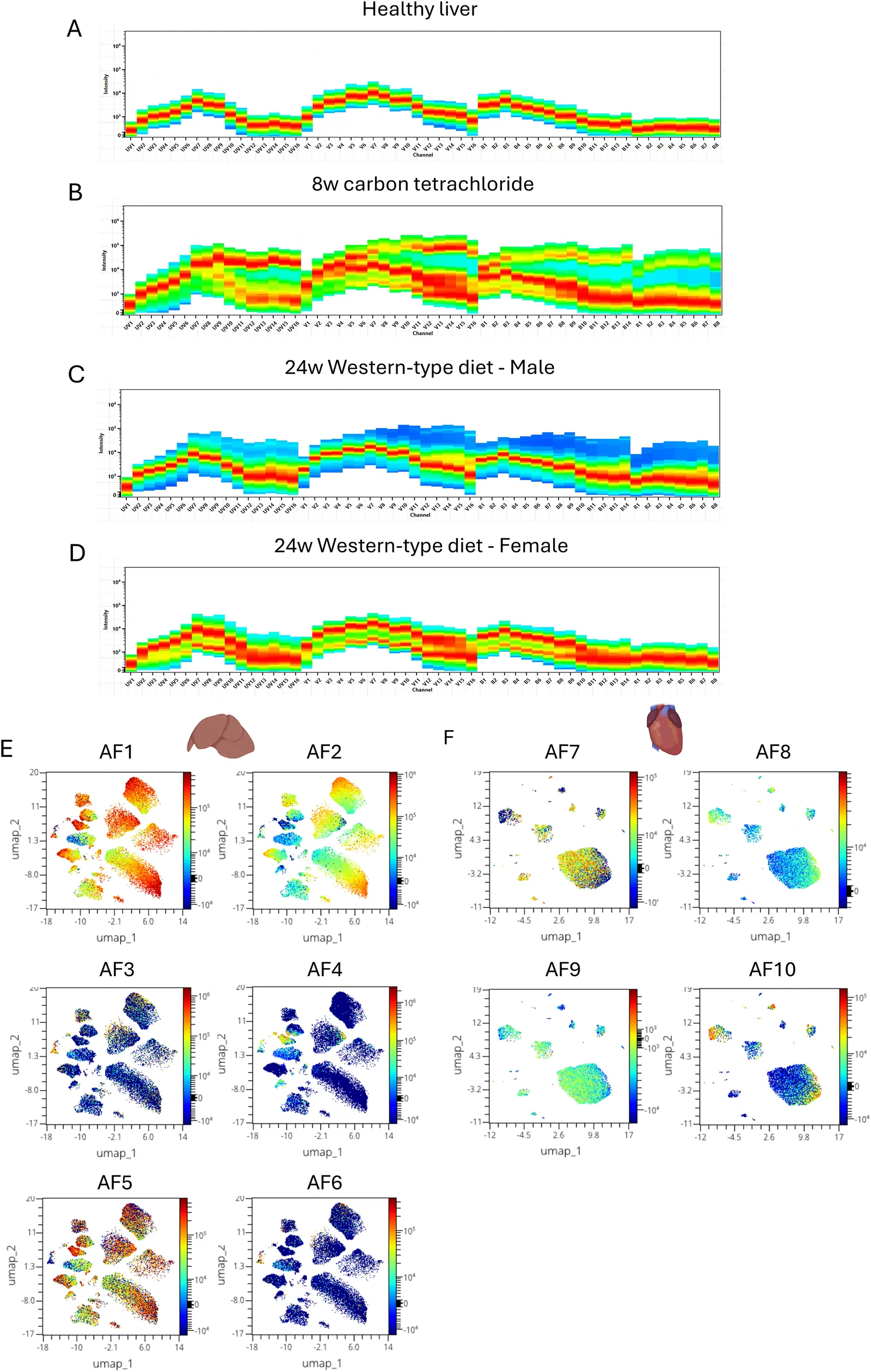
**Examples of autofluorescence signatures in different murine liver samples.** Spectral plots of unstained samples obtained from (A) healthy male liver tissue, (B) fibrotic liver tissue after 8 weeks of carbon tetrachloride injections in a male mouse, (C, D) steatotic liver after 24 weeks of a Western-type diet in (C) a male mouse and (D) a female mouse. The high diversity demonstrates the requirement for group-specific unmixing. (E, F) Heatmaps of autofluorescence intensity and distribution across the different (E) hepatic and (F) cardiac endothelial cell clusters.

### 3.4 Additional controls and data analysis

#### 3.4.1 Fluorescence Minus One (FMO) control samples

To establish a threshold for positive signal for each target, it is essential to include appropriate FMO controls in the experiment. As discussed previously, fluorochrome spread can increase background signal, making it difficult to distinguish true positive signal from noise. FMO controls address this by using all fluorochromes in the panel but one for each marker of interest. This approach provides a clear background threshold and defines the point beyond which a positive gate should be drawn for each fluorochrome (Fig. 10A–N).

**Fig. 10:**
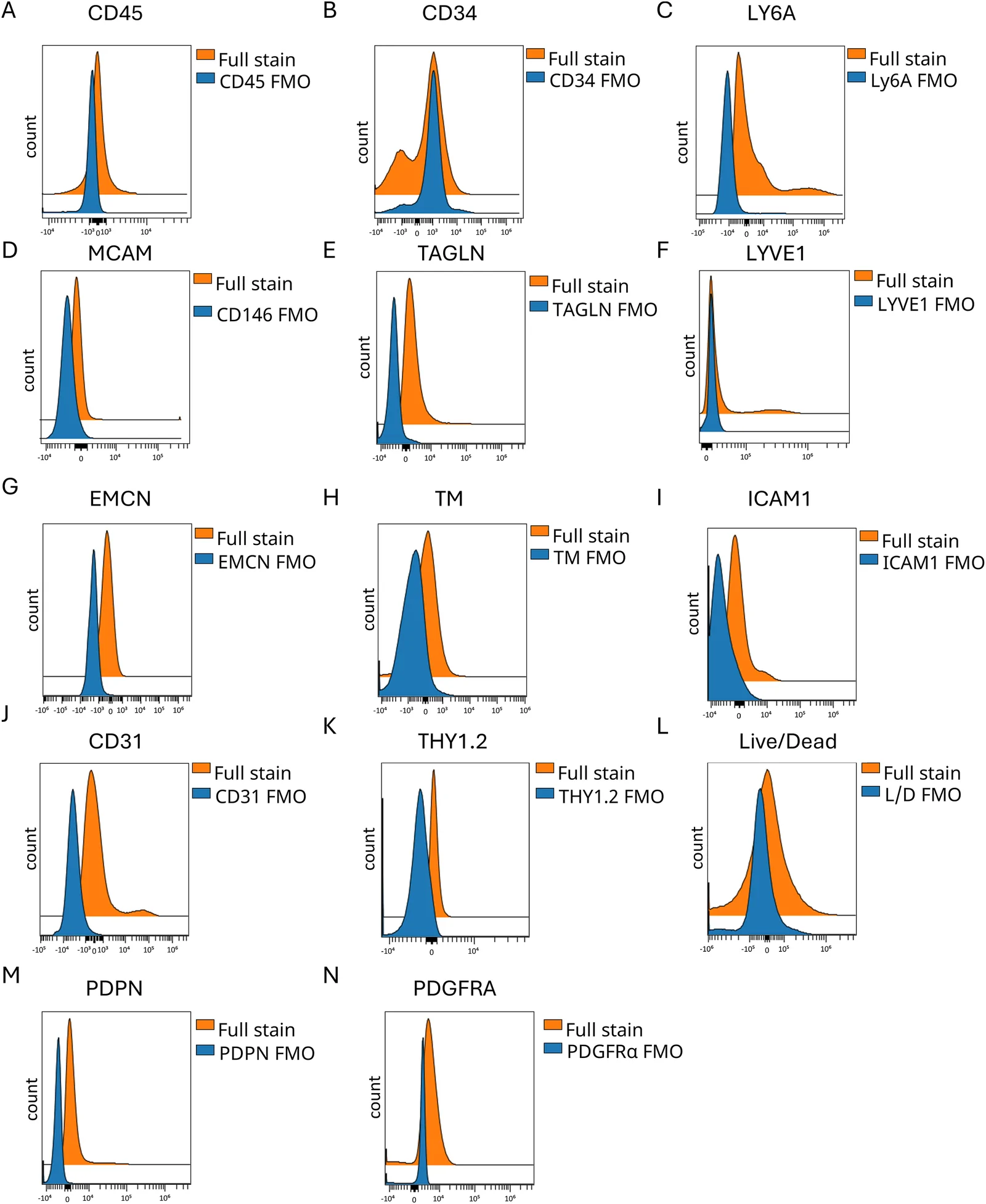
**FMO controls for all markers of the EC panel.** Histograms comparing Fluorescence Minus One (FMO; blue) samples to full stain samples (orange) in heathy liver cells for (A) CD45, (B) CD34, (C) LY6A, (D) melanoma cell adhesion molecule (MCAM), (E) transgelin (TAGLN), (F) LYVE1, (G) endomucin (EMCN), (H) thrombomodulin (TM), (I) ICAM1, (J) CD31, (K) THY1.2, (L) LIVE/DEAD Green Fixable dead cell dye, (M) podoplanin (PDPN), (N) platelet-derived growth factor receptor α (PDGFRα).

#### 3.4.2 High dimensionality reduction

In addition to traditional gating analysis, FSFC allows for the use of dimensionality reduction analysis. Given the complexity of the panel and the diversity of cell phenotypes studied, clustering analysis can be used to identify cells sharing multiple properties, rather than relying on conventional manual NxN plots that analyse only two parameters at a time.

Because signal acquisition can be affected by several factors such as the presence of air bubbles, laser and instrument fluctuation or changes in flow rate, a QC step is essential to ensure data integrity. Various algorithms are available for QC; in our workflow we applied FlowAI [11] to identify and remove anomalous data acquired through variation in the flow rate and signal range over time. Importantly, uncompensated data should be used for this algorithm, allowing for unbiased QC, not affected by user-dependent manual compensation. FlowAI generates a new gate containing ‘clean’ data, which is then used for all downstream analyses.

After QC, data were properly scaled and compensated, before being manually cleaned to remove debris, doublets and dead cells (Fig. 6A–C). To identify unique EC subpopulations, we performed unsupervised clustering based on the 6 EC identity markers of the panel. Data from all experimental groups were concatenated prior to clustering, allowing subsequent analysis and comparison between samples groups. Clustering was undertaken using the FlowSOM [12] algorithm, which generates a self-organizing map that uses K nearest neighbours, where K is manually defined by the user. We selected K = 20, anticipating more than 5 but fewer than 20 subpopulations (meta-clusters), based on previous scRNAseq studies [17], [18]. Once clusters were generated, their properties were reviewed manually using the MFI of their positive markers, and clusters were manually merged or split as needed using Boolean filters. To assess the robustness of the unsupervised clustering, we compared K = 10, 20 and 30. At K = 10, only six clusters were identified, indicating under-clustering and insufficient resolution to capture the heterogeneity of the dataset. In contrast, both K = 20 and K = 30 resolved 12 biologically distinct clusters following manual consolidation, supporting K = 20 as an appropriate choice for the analysis. The majority of clusters identified across all three K values were consistent, with comparable relative frequencies (Fig. S4), while some variability in cluster proportions was observed, as expected with changes in clustering number and parameters.

Dimensionality reduction plots can then be used to visualize the clusters generated. Depending on the data and biological questions, different types of plots can be used and the settings for map generation can be optimized to better present the data. Clustering data for hepatic and cardiac EC subpopulations (Fig. 11A and B respectively) were visualized by Uniform Manifold Approximation and Projection (UMAP) plots [13]. UMAP better preserves the global structure of the data allowing to clearly segregate each cluster and thereby visualize their expression profiles for each EC marker within the map (Fig. 11C and D). Whilst the same EC panel can be applied across different tissues, clustering analysis must remain tissue-specific to accurately capture differences in marker expression profiles. For this reason, each UMAP visualization was generated separately for each tissue type, and the UMAP plots cannot be directly overlaid. However, UMAP plots highlight the greater diversity of hepatic EC subpopulations compared to those in the heart, with 8 of the 12 hepatic clusters expressing more than 4 EC identity markers, compared to only 1 of 7 within the heart. Additionally, the composition of heart EC is dominated by a single, large subpopulation while hepatic subpopulations are more evenly distributed between the clusters (Fig. 11E and F). To confirm that the high dimensional reduction pipeline can be validated by a conventional gating strategy we present both dot plots and a schematic gating map for each of the EC markers for both liver (Fig. S5 A&B) and heart (Fig. S6 A&B) EC clusters.

**Fig. 11:**
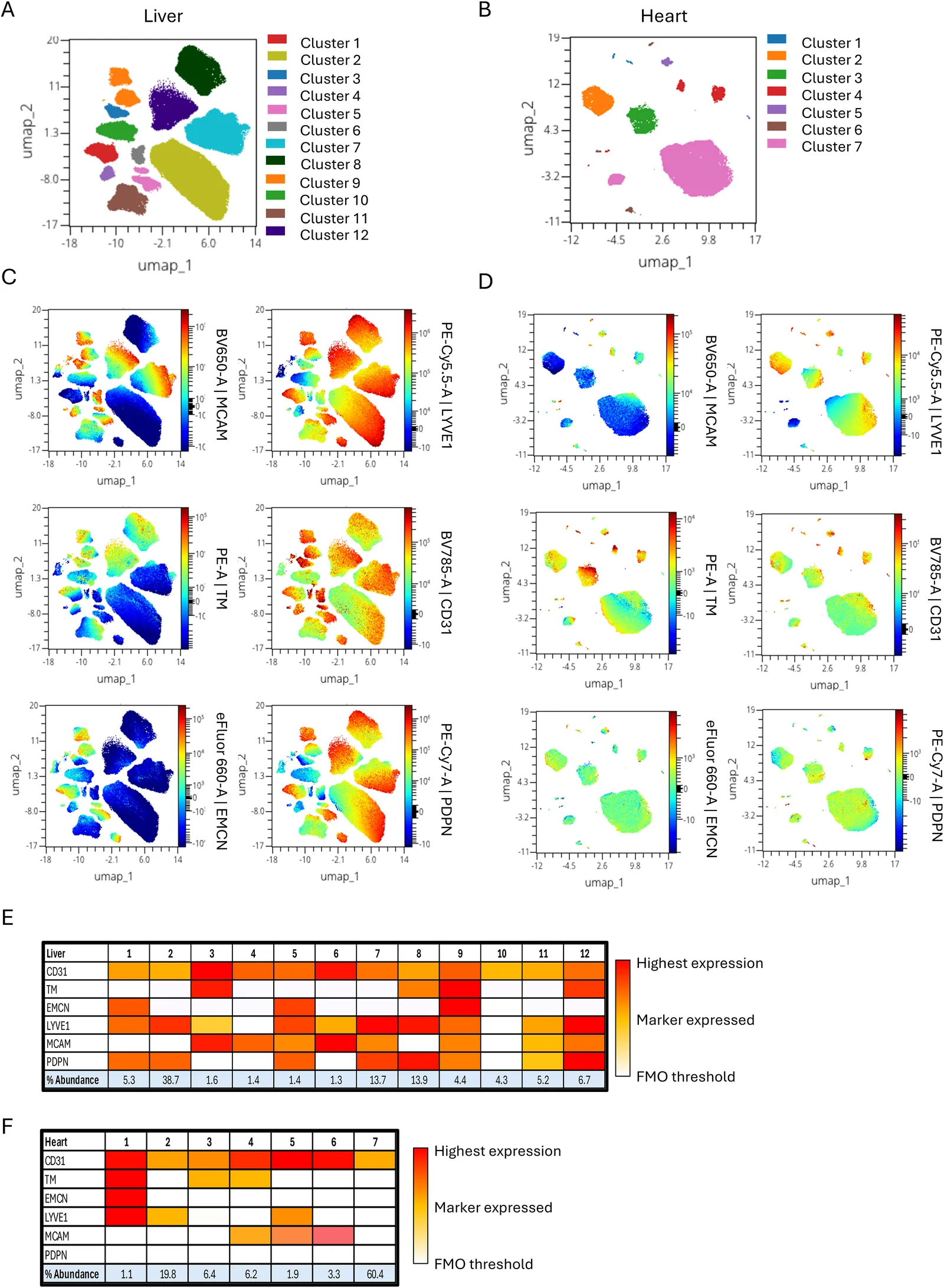
**Dimensionality Reduction analysis of hepatic and cardiac endothelial cells.** (A, B) UMAP plots generated using the endothelial markers CD31, thrombomodulin (TM), endomucin (EMCN), LYVE1, melanoma cell adhesion molecule (MCAM), and podoplanin (PDPN) for CD31^+^CD45^−^ endothelial cells from healthy murine (A) liver and (B) heart tissue. (C, D) Expression of each marker was overlaid onto the UMAP to distinguish the characteristics of each cluster for (C) liver and (D) heart samples. (E, F) Heatmaps of the mean fluorescence intensity of each EC identity marker used to define the 12 hepatic EC clusters (E) and the 7 cardiac EC clusters (F). The colour denotes the intensity of expression of a marker; below the FMO threshold (white), expression above the FMO threshold (yellow) and highest expression (red). The abundance of each cluster expressed as a percentage of the total EC is also presented.

#### 3.4.3 Application of secondary analytical tools to EC subpopulations

As our experimental approach included multiple timepoints and measured markers of activation status, we had the opportunity to assess cellular dynamics by modelling their trajectory. This was achieved by applying Wishbone trajectory analysis which has previously been published to infer T-cell differentiation. Wishbone enables an unbiased assessment of cell plasticity from a manually identified starting cell population [14]. In this example we began with EC that had low activation status defined as ICAM1^-^ TAGLN^−^ (Fig. 12A). To determine the trajectory of activation of these cells we used Wishbone analysis which identified a trunk and two branches (Fig. 12B) based on the changes in expression of ICAM1, LY6A, CD34, TAGLN, THY1.2 and PDGFRα. The Wishbone algorithm allocates ‘milestones’ for each cell analysed, building a trajectory that can be linear, or include a bifurcation point [31], providing insight into lineage relationships [10]. These criteria enabled us to visualize the phenotypic changes that occur over time in response to Western diet (Fig. 12C). Wishbone analysis represents an exciting retrospective approach to tracking cellular plasticity using proteomic data and would offer new dimensions to lineage tracing approaches.

**Fig. 12:**
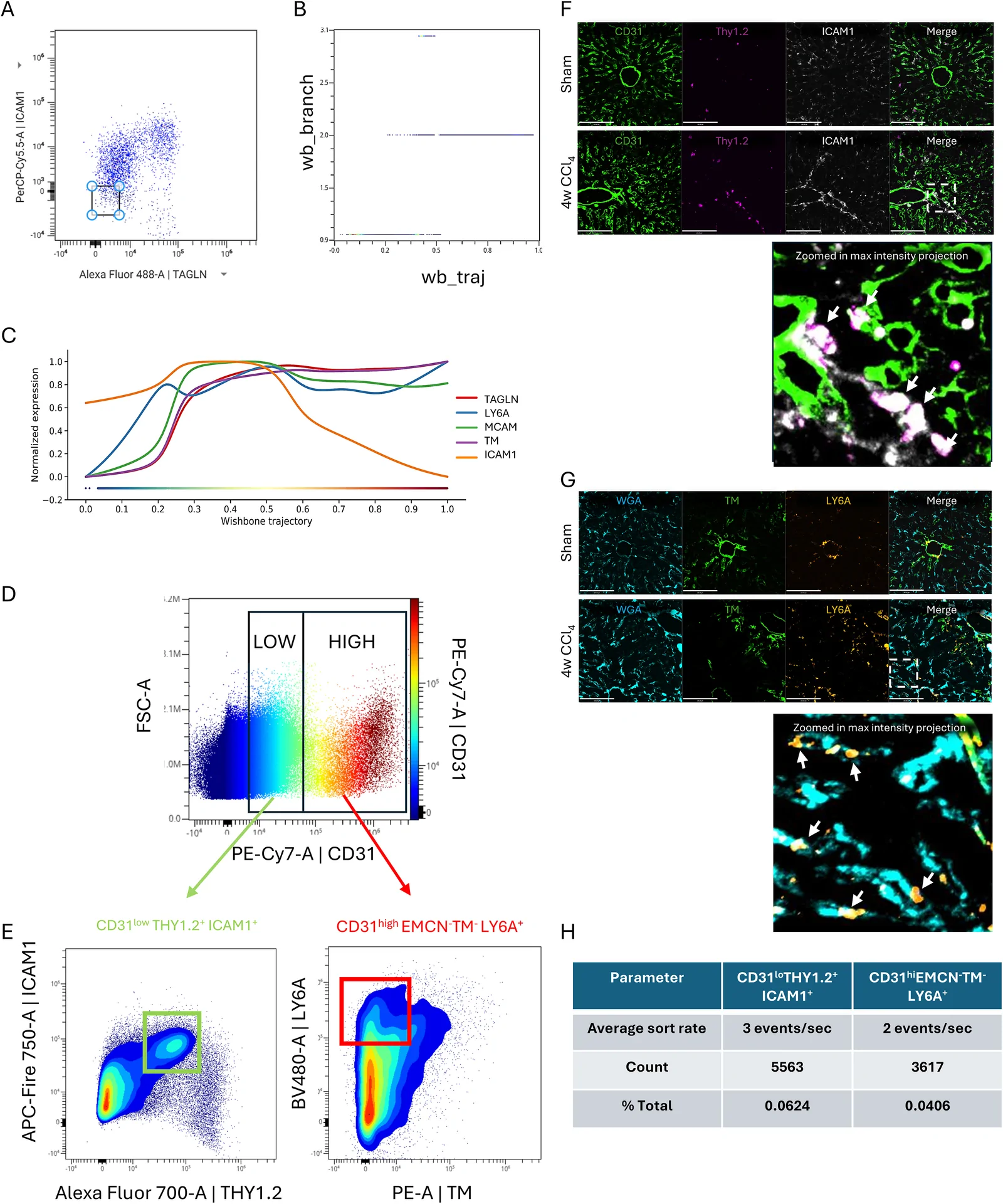
**Secondary analysis of EC subpopulations by Wishbone trajectory and full spectrum cell sorting.** (A-C) Example workflow for generating a Wishbone trajectory plot. (A) Dot plot of TAGLN vs ICAM1 to identify the starting gate for Wishbone analysis, defined as TAGLN^−^ ICAM1^-^ cells. (B) Branch and trajectory plot which allocates milestones for each cell analysed. (C) Wishbone trajectory plot for TAGLN, LY6A, MCAM, TM and ICAM1 highlighting the transition of EC in response to 12w Western diet. (D) Dot plot of CD31 expression in whole liver cell suspension from mice treated with carbon tetrachloride for 4 weeks. (E) Full spectrum cell sorting of CD31^low^ THY1.2^+^ ICAM1^+^ (left; green gate) and CD31^high^ TM^−^LY6A^+^ (right; red gate). Representative confocal images of IF staining for (F) CD31 (green) and THY1.2 (magenta) and ICAM1 (white) and (G) CD31 (green) and LY6A (orange) and wheat germ agglutinin (WGA; cyan) in Sham and 4w CCl4-treated mice with a zoomed in region of a maximum intensity project including white arrows highlighting each EC subpopulation present below each panel. Scale bar = 100 μm. (H) Table of sorting efficiency parameters for each population.

Using our initial EC panel, we demonstrated changes in inflammatory status and induction of Endothelial-to-Mesenchymal Transition (EndMT) associated with murine models of progressive liver fibrosis [10]. This comprehensive phenotyping approach allowed the isolation of rare and complex EC subpopulations through full spectrum cell sorting. Thus, we identified a pro-fibrotic EC phenotype, expressing the EndMT marker THY1.2, which was preferentially expressed on CD31^low^ ECs. In contrast, a LY6A^+^, pro-inflammatory EC phenotype was preferentially acquired by CD31^high^ ECs (Fig. 12D and E) after 4 weeks of carbon tetrachloride (CCl_4_)-induced liver fibrosis. The induction of THY1.2^+^ ICAM1^+^ and LY6A^+^ TM^−^ EC subpopulations following 4w CCl_4_ treatment was confirmed by IF (Figs. 12F and G respectively). These findings informed the development of a targeted cell-sorting panel which was transferable to the Cytek® Aurora CS cell sorter system (Table 3) with the help of Cytek®. While antibody clones remained unchanged, the panel was streamlined to reduce complexity and improve separation, thereby enhancing sort efficiency and resolution of these rare EC subpopulations (Fig. 12H).

**Table 3:** Antibody panel for full spectrum cell sorting.

REAGENT or RESOURCE	SOURCE	IDENTIFIER
Antibodies		
CD31 (Clone 390) PE-Cy7	Biolegend	102417
CD45 (Clone 30-F11) BUV395	BD Bioscience	565613
Thrombomodulin (Clone LS17-9) PE	Fisher Scientific	12-1411-82
EMCN (Clone V.7C7) eFluor660	Fisher Scientific	50-5851-82
LYVE1 (Clone ALY7) PE-Cy5.5	Novus	NBP1-43411
LY6A (Clone D7) BV480	Biolegend	746663
ICAM1 (Clone YN1/1.7.4) PerCP-Cy5.5	Biolegend	116123
THY1.2 (Clone 30-H12) AF700	Biolegend	105320
CD34 (Clone MEC14.7) BV421	Biolegend	119321

## 4 Discussion

Flow cytometry techniques have provided remarkable insight into the identity and phenotype of circulating cell types, such as leukocytes [32], platelets [33] and cancer cells [34], and have become a gold standard technique in immuno-oncology [2]. The iterative process of acquiring and identifying new cellular phenotypes has driven the emergence of new technologies, such as FSFC, capable of resolving more than 50 fluorochromes [35]. In this study we exemplify how this technology is not restricted to profiling non-adherent cells, but can be a powerful technique to assess cells derived from solid tissue which traditionally has been limited due to several challenges. These include tissue dissociation, complex tissue- and cell-specific AF signatures and availability of appropriate antibody reagents. To address this knowledge and application gap we established a 14-colour EC panel designed to identify specific EC subpopulations and assess phenotypic changes induced in response to tissue fibrosis. Although ECs are highly diverse, as shown in numerous scRNAseq studies [4], [5], [6], [36], proteomic phenotyping remains comparatively underexplored. We aimed to integrate our knowledge of vascular heterogeneity in health and disease to identify key markers of EC identity, inflammation and fibrosis using commercially available antibodies to develop a framework for an EC-specific FSFC panel.

ECs represent a particularly challenging population for flow cytometry due to their high and complex AF signatures [7]. We addressed these hurdles by incorporating a multitude of controls to identify confounding factors that influence spectral unmixing. Notably, healthy livers displayed between 3 and 4 AF signatures, whereas mice treated for 8 weeks with CCl_4_ had up to 7 distinct AF signatures, significantly increasing the complexity score of the panel. Initially, model- and time-point-specific AF signatures required laborious manual identification and removal. However, the release of the Cytek® Multi-AF extraction tool in 2024 has dramatically simplified multi-AF identification and improved its removal within an unmixing workflow.

Our core EC identity markers displayed remarkable diversity across the EC subpopulations, enabling the resolution of 12 EC subpopulations in the liver and 7 within the heart. These markers represent diverse aspects of EC identity and are associated with a variety of biological functions [37]. Although direct comparison between liver and heart EC populations is challenging due to their distinct physiological roles, we observed clear tissue-specific phenotypical differences. For example, LYVE1 and PDPN expression was far more prominent in the liver compared to the heart, reflecting the specialized nature of hepatic ECs, which share traits with lymphatic vessels [38]. Furthermore, the inclusion of activation markers enabled the identification of phenotypical differences in liver ECs in a murine model of liver fibrosis, revealing changes associated with inflammation and fibrosis-induced EndMT [10]. We also showed that Wishbone analysis can provide novel insights into EC plasticity and state transition. Finally, we demonstrated that these unique phenotypical features can be leveraged for full spectrum cell sorting, enabling isolation of rare EC subpopulations for further downstream functional and molecular analysis.

In summary, we demonstrated the successful application of an EC-specific FSFC panel to liver and heart tissues and its utility in models of liver injury. This work establishes a robust methodological foundation that will accelerate high-dimensional phenotyping of complex cell populations and expand the capabilities of FSFC in solid tissue research.

### 4.1 Limitations of the study

The FSFC protocol described has several inherent limitations. As experiment-specific AF controls are required for accurate unmixing, variability between datasets can be introduced, necessitating larger biological sample numbers to achieve robust and reproducible results. Moreover, unlike scRNAseq, which enables unbiased discovery of transcriptional states, FSFC-based cluster identification is constrained by the markers included in the antibody panel. The limited choice of fluorochrome conjugates of non-immune cell targets is an important challenge that, if addressed, could unlock the discovery of rare or unexpected cell phenotypes. It is important to note that this workflow should be used as an example for applying FSFC to solid tissue and should be adapted as appropriate based on the experimental conditions, AF signatures and antibody panel.

Using this technology we have shown complementary results with immunofluorescence and scRNAseq data [10] however, we have not assessed if the EC subpopulations we describe can be recapitulated with non-fluorescent based approaches, such as CyTOF, which would be an important comparison to verify the conservation of EC subpopulations between different platforms and determine the full impact of autofluorescence in future studies.

## CRediT authorship contribution statement

**Christina Gkantsinikoudi:** Writing – review & editing, Writing – original draft, Visualization, Methodology, Investigation, Formal analysis, Data curation, Conceptualization. **Manuela Terranova-Barberio:** Writing – review & editing, Writing – original draft, Supervision, Methodology, Conceptualization. **Neil Dufton:** Writing – review & editing, Writing – original draft, Visualization, Supervision, Project administration, Investigation, Funding acquisition, Data curation.

## Motivation

Single-cell RNA sequencing has transformed our understanding of cellular heterogeneity, but transcriptomic data alone cannot capture information regarding protein expression, post-transcriptional modifications or cell-surface interactions. Full spectrum flow cytometry (FSFC) offers high-dimensional proteomic analysis at single-cell resolution, yet its application to non-immune cells, particularly endothelial cells (EC) from solid tissues, is limited due to their complex autofluorescence signatures. We developed and optimized a FSFC protocol to overcome this challenge, enabling robust phenotyping of endothelial subpopulations, downstream high dimensional analysis and isolation of rare EC subpopulations from murine liver and heart tissues.

## Declaration of competing interest

The authors declare that they have no known competing financial interests or personal relationships that could have appeared to influence the work reported in this paper.

## Data Availability

The datasets from the current study are available from the corresponding author on reasonable request.
