# Supplementary material for: Addressing complex autofluorescence signatures in solid tissue samples to enhance full spectrum flow cytometry of non-immune cells
Source: Biochem Biophys Rep. 2026 Oct 17;48:102845. doi: 10.1016/j.bbrep.2026.102845 (PMC13696768; doi:10.1016/j.bbrep.2026.102845)

Supplementary Figure 1

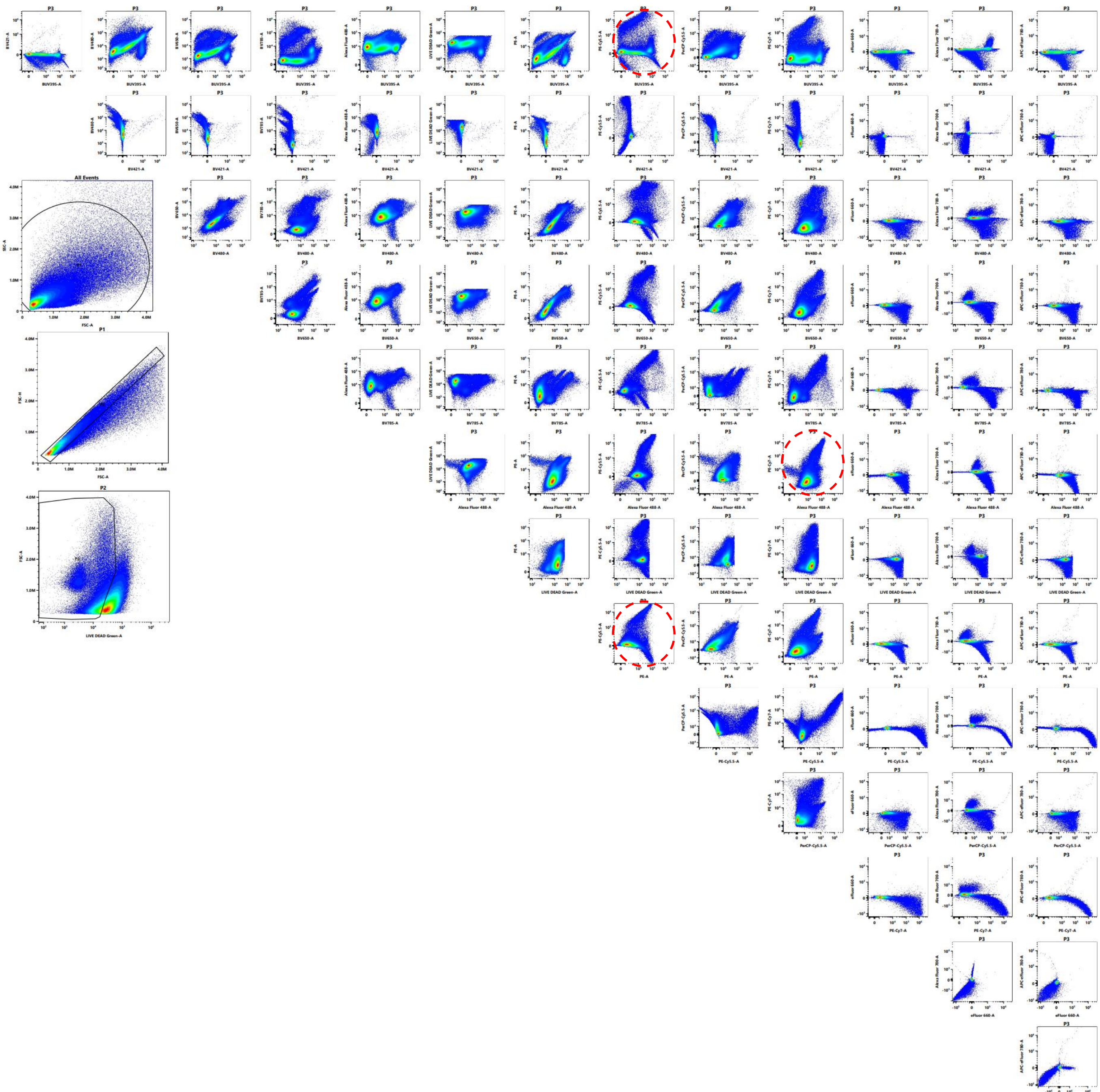

Supplementary Figure 2

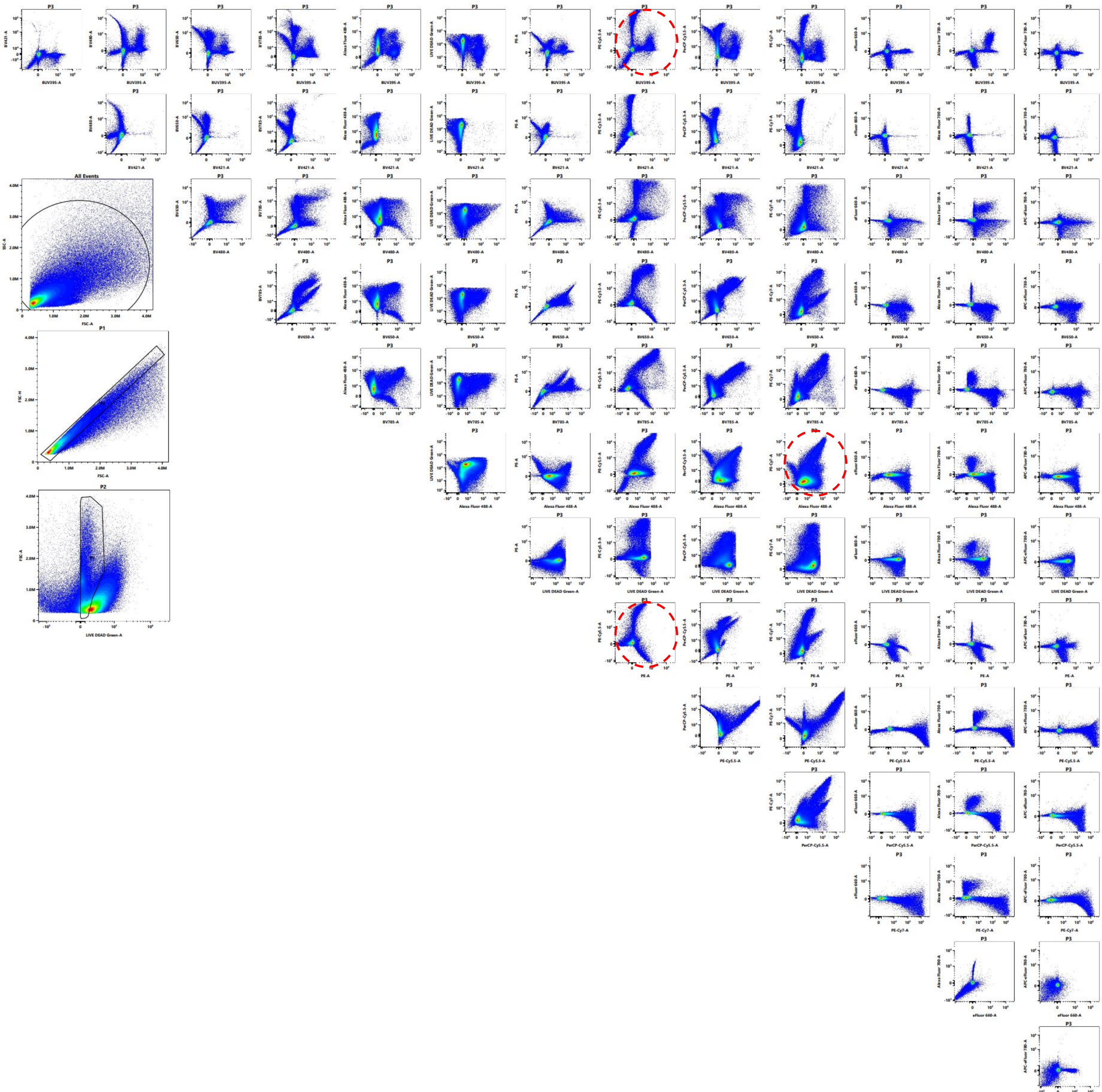

Supplementary Figure 3

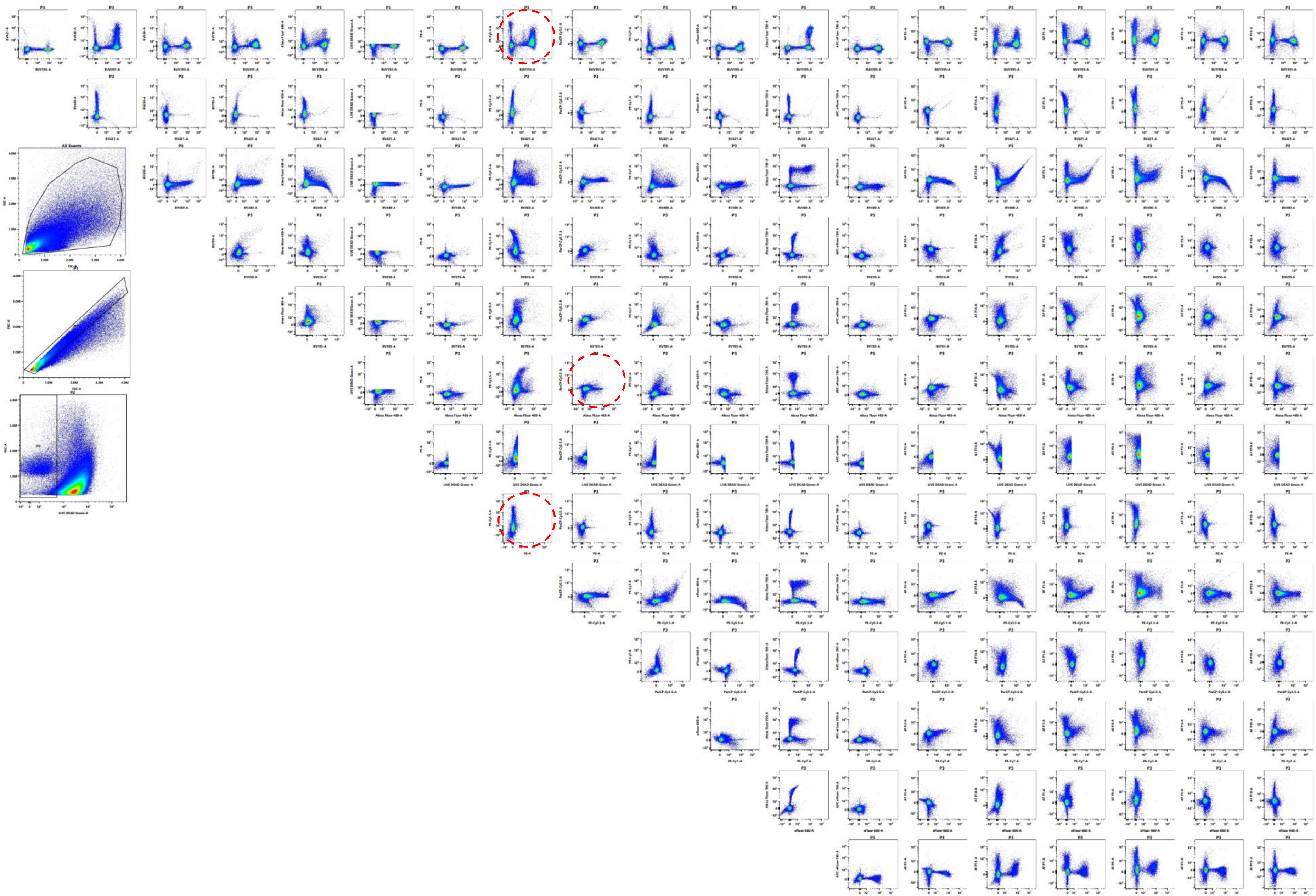

Supplementary Figure 4

A

Liver	1	2	3	4	5	6	7	8	9	10	11	12	13	14	15
CD31															
MCAM															
TM															
LYVE1															
PDPN															
EMCN															
**% Abundance**	7.3	51				20.1			5				14.6	1.4	

B

Liver	1	2	3	4	5	6	7	8	9	10	11	12	13	14	15
CD31															
MCAM															
TM															
LYVE1															
PDPN															
EMCN															
**% Abundance**	5.3	38.7	1.6	1.4	1.4	1.3	13.7	13.9	4.4	4.3	5.2	6.7			

C

Liver	1	2	3	4	5	6	7	8	9	10	11	12	13	14	15
CD31															
MCAM															
TM															
LYVE1															
PDPN															
EMCN															
**% Abundance**	3.6	42.7		1.9	1.6	17.2	4.8	12.1	2.4	2.7		4.8		1.9	2.8

Supplementary Figure 5

A

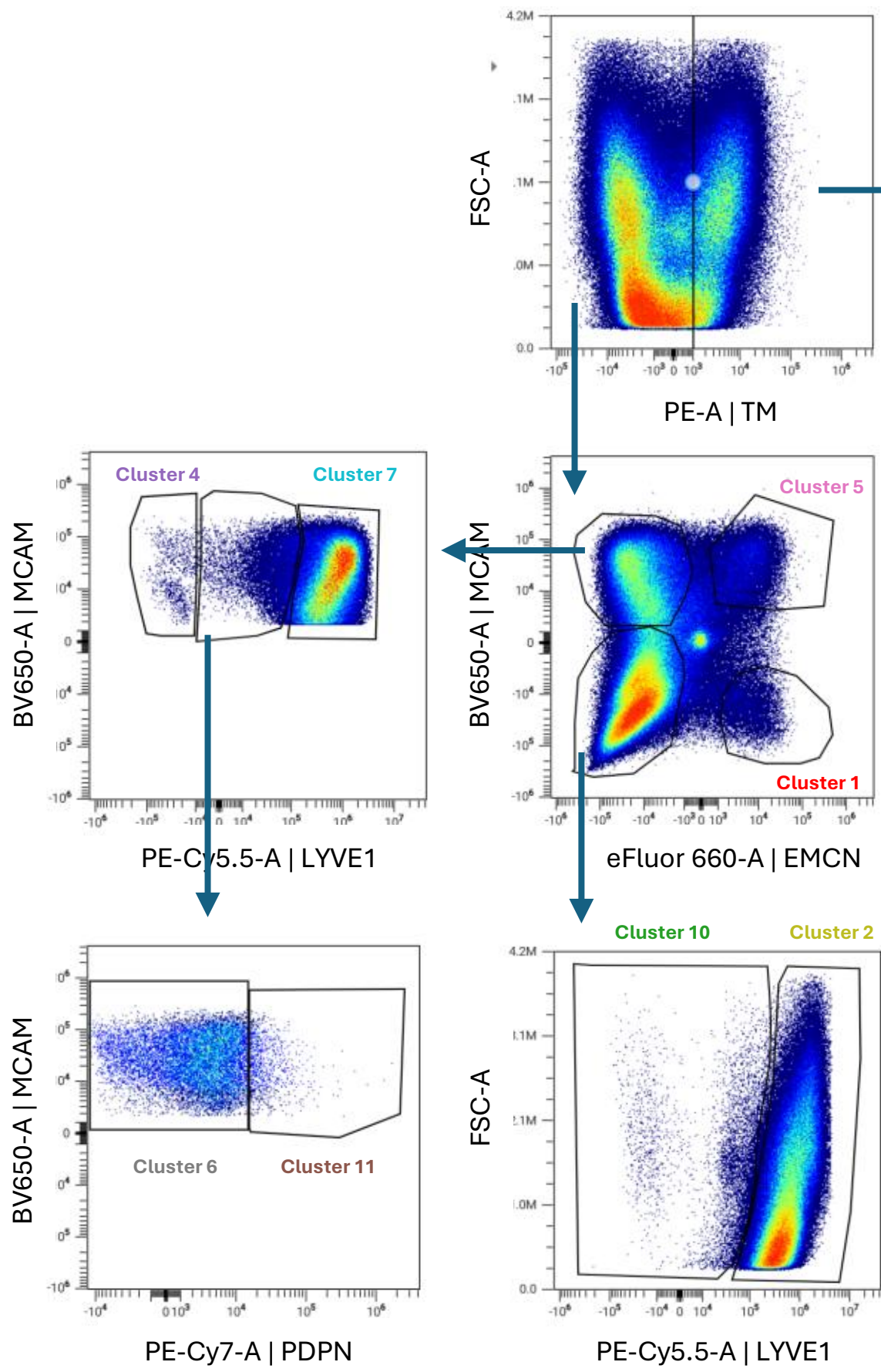

B

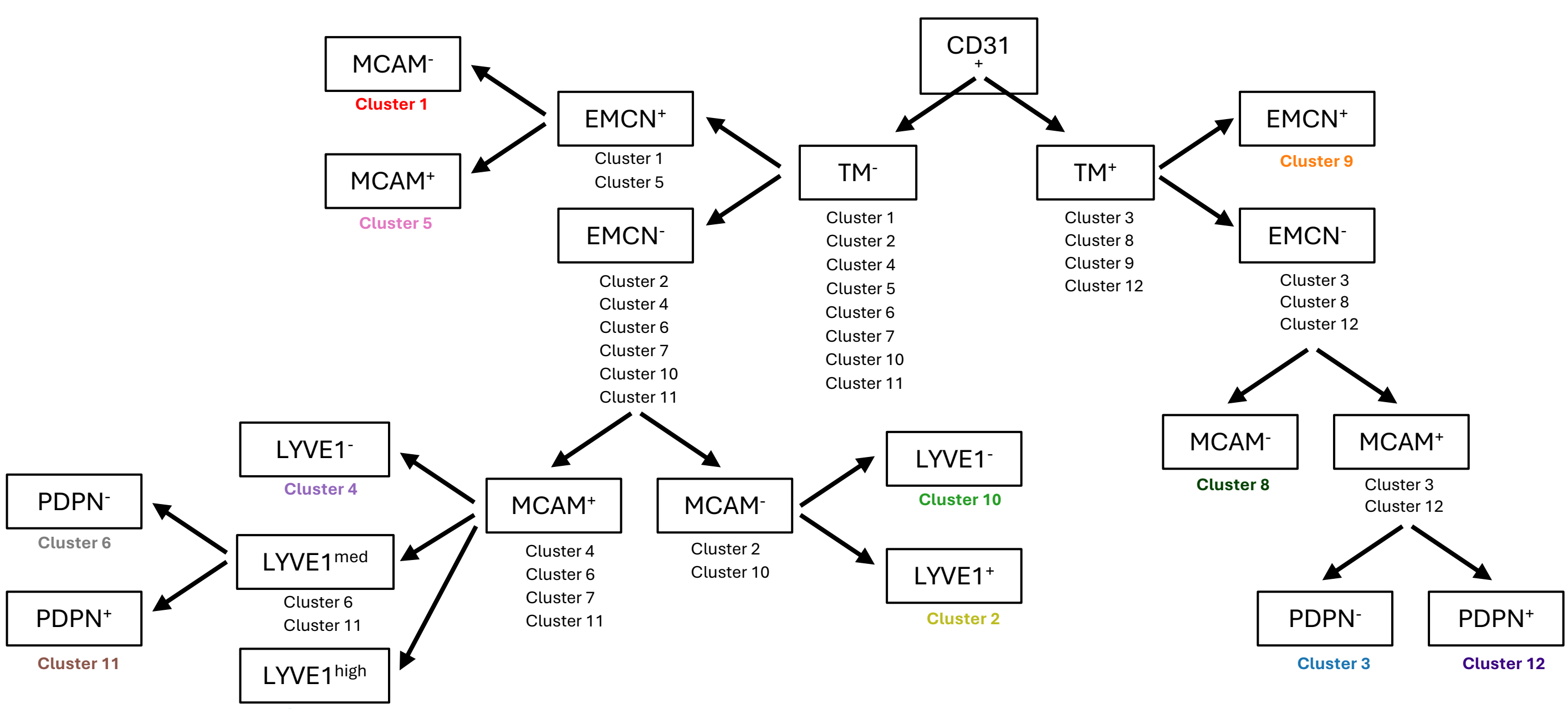

Supplementary Figure 6

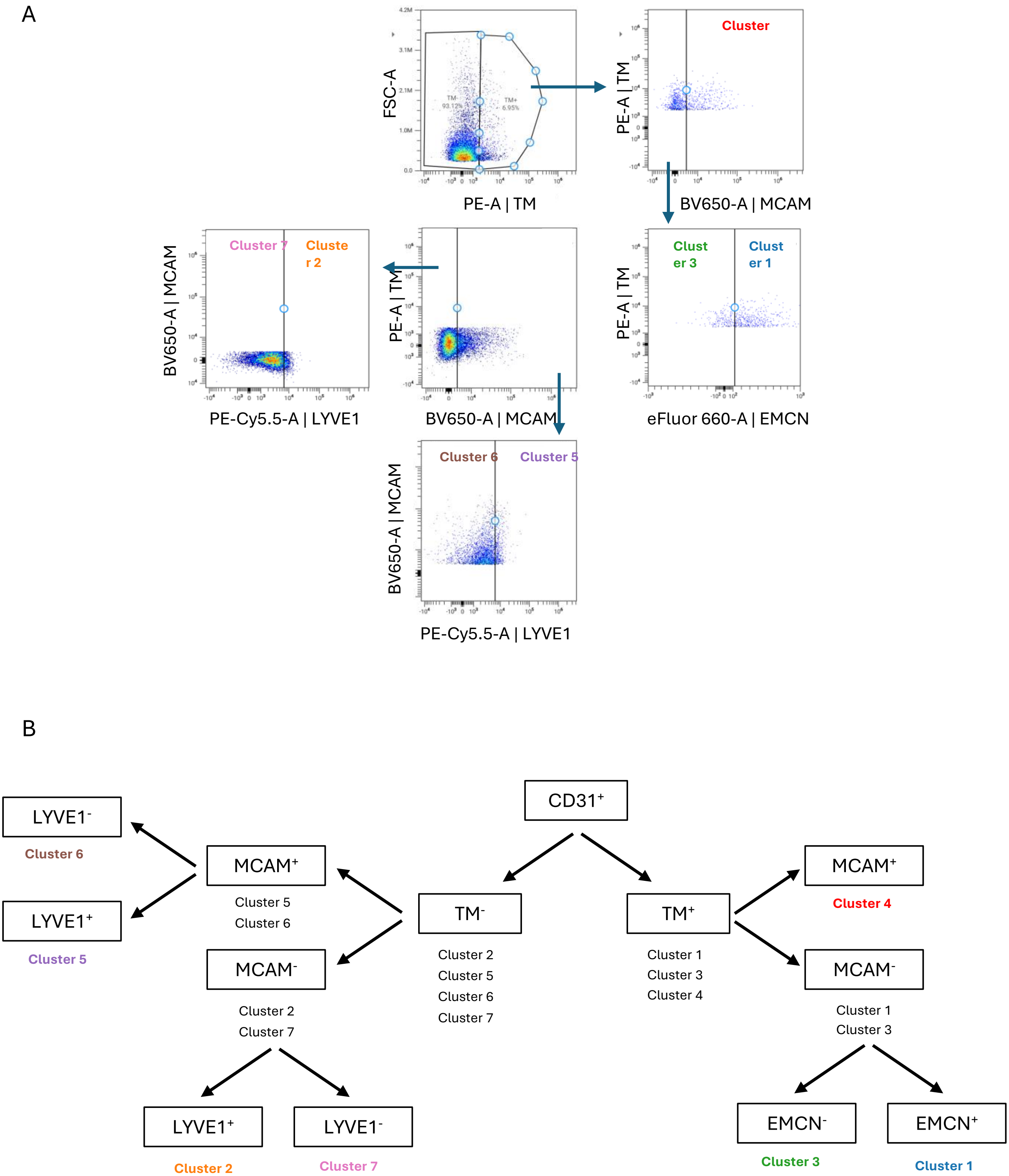

Supplement: Supplementary file 1 [file mmc1.pdf]
